# Medication Adherence Following Stroke and TIA: A Qualitative Synthesis of Patient, Caregiver and Clinician Perspectives

**DOI:** 10.3390/neurolint18020034

**Published:** 2026-02-11

**Authors:** Erin O’Kane, Rhiannon De Ivey, Katie Pearson, Christa Awad, Khalifa Mohammed, Nathan Williamson, Richard Andrew Lumb, Ami Mehta, Eugene Yee Hing Tang

**Affiliations:** 1 School of Medicine, University of Sunderland, Sunderland SR1 3SD, UK; 2 Population Health Sciences Institute, Newcastle University, Newcastle upon Tyne NE1 7RU, UK; 3 Edinburgh Medical School, University of Edinburgh, Edinburgh EH16 4UX, UK; 4 The Newcastle upon Tyne Hospitals NHS Foundation Trust, Newcastle upon Tyne NE1 4LP, UK; 5 Walton Library, Newcastle University, Newcastle upon Tyne NE2 4HH, UK; 6 School of Medicine, Newcastle University, Newcastle upon Tyne NE2 4HH, UK

**Keywords:** stroke, adherence, TIA, medication, systematic review

## Abstract

**Background/Objectives**: Stroke survivors require life-long secondary prevention to reduce recurrence, but they also often face long-term impairments that may limit medication adherence (MA) including cognitive, physical, and psychological effects. This updated qualitative meta-synthesis aims to descriptively explore and synthesise the experiences and perspectives of stroke/TIA survivors, informal and formal carers of stroke survivors, and healthcare professionals involved in post-stroke/TIA care, with a focus on factors influencing and hindering MA. **Methods**: A qualitative meta-synthesis was conducted in accordance with PRISMA guidance. Searches were undertaken across MEDLINE, CINAHL, Embase, PsycINFO, Scopus and Web of Science for studies published from 1 January 2018. Study quality was assessed using the Joanna Briggs Institute checklist and data synthesised using Thomas and Harden’s method. **Results**: Of 5463 titles and abstracts screened, 212 underwent full-text review with 13 papers meeting inclusion criteria from eight countries with a total of 435 participants. Seven key themes were identified: knowledge and understanding, beliefs and attitudes, practical barriers, social support, healthcare system, psychological factors and medication characteristics. Survivors showed a varied understanding of their condition and prescribed medicines, with unclear communication often contributing to confusion. Beliefs and attitudes shaped adherence, ranging from confidence in treatment to scepticism. Practical barriers included financial costs, physical impairments, and limited access to services. Social support from family, friends, and healthcare professionals was also important. Psychological wellbeing, coping strategies, and medication side effects further influenced adherence, highlighting the challenges faced by this patient group. **Conclusions**: Medication adherence post-stroke/TIA is shaped by multiple complex factors including knowledge, beliefs, attitudes, and lived experience. As a descriptive synthesis of qualitative evidence, these findings do not permit conclusions regarding causality or intervention effectiveness but provide insight into perceived barriers and facilitators that may inform future intervention development and clinical questioning.

## 1. Introduction

Stroke and transient ischaemic attack (TIA) affect 12 million people globally each year [1]. Older patients are particularly affected, where the complexity of care and associated healthcare costs place a significant burden on individuals and health systems alike [1]. Nearly one in five stroke survivors will have a recurrent stroke within 5 years [2]. To counteract this, both stroke and TIA patients require life-long secondary prevention medication to reduce this risk. Despite patients receiving education through, for example, medication reviews, the overall medication adherence and persistence rates are low in patients who have had a stroke [3].

There are many possible reasons for this, including that stroke-survivors often face long-term functional (and hidden) impairments that may create practical challenges for medication adherence (MA). This can include, for example, cognitive impairment, which can limit their ability to manage complex medication regimens. Post-stroke cognitive impairment is recognised as a major source of morbidity post-stroke and includes impaired attention, memory and executive functioning, all of which may affect the individual’s ability to adhere to medication regimes post-stroke [4]. Limitations in motor function may cause difficulties with tasks such as opening and handling medication, while psychological effects such as depression may reduce motivation to maintain consistent routines [5]. Further, limited public awareness and understanding of these conditions may contribute to patient non-adherence, as stroke and TIA survivors may underestimate the severity and implications of the condition [6].

To effectively respond to this issue, it is important to identify factors that hinder and support MA among stroke and TIA survivors. Current research with older adults shows that to improve MA, there is a need to evaluate strategies tailored to the patient experience [7]. In the context of stroke and TIA, patients will also commonly have multiple comorbidities, whilst also needing to adapt to their physical and hidden post-stroke impairments. There have been some organisational interventions which have been associated with improving blood pressure targets but no clear evidence on any other modifiable risk factors, which include lipid profile and medication adherence [8]. These interventions, that generally include patient education, were not found to prevent recurrent cardiovascular events [8] which further emphasises the need to understand what is needed to improve MA. A meta synthesis, published in 2019, examined the perspectives of both survivors and carers around MA [9]. Here, they concluded that the burden of “medicines work” after stroke is substantial and multifaceted with many studies displaying methodological weaknesses [9]. Since then, there has been further emerging research in this area warranting an updated synthesis to capture developments beyond this period. This is particularly relevant given that healthcare systems, such as the National Health Service in England, are advocating for a shift towards disease prevention in the community [10].

The aim of this review was to undertake an updated meta-synthesis exploring the experiences and perspectives of stroke and/or TIA survivors in relation to medication adherence.

## 2. Materials and Methods

An updated review was conducted in alignment with the Preferred Reporting Items for Systematic Reviews and Meta-Analyses (PRISMA) guidance [11]. The PRISMA checklist is included in Supplementary S5. This qualitative meta-synthesis did not require ethical approval or informed consent and was registered with PROSPERO (CRD420251053596). 

### 2.1. Search Strategy

A search was conducted across various databases including MEDLINE, CINAHL, Embase (via Ovid), PsycINFO, Scopus, and Web of Science. The search was limited to studies published from January 2018 to update the original review [9]. Additional methods such as reference list screening of included studies were also carried out to identify any relevant papers not captured through database searching. The full search strategy is publicly available via PROSPERO (CRD420251053596) and Supplementary S1. The screening and data management process was carried out using Covidence software (https://www.covidence.org/terms/, accessed on 26 January 2026). The most recent search was conducted up to 15 May 2025.

### 2.2. Eligibility Criteria

Studies were included if they involved stroke or TIA survivors, informal or formal carers of stroke survivors, or healthcare professionals such as general practitioners, stroke specialists, nurses and pharmacists involved in post-stroke or TIA care. To be eligible, participants must have been prescribed or involved in the prescription, supply or support of medication for secondary prevention or management of co-morbidities following stroke or TIA. Only qualitative or mixed-method studies reporting qualitative and quantitative data separately were considered. Due to the qualitative meta-synthesis approach, studies had to be published in English and in full-text format. Non-randomised study types were eligible for inclusion.

Studies were excluded if they involved populations unrelated to stroke or TIA, or if they lacked a qualitative component. Mixed-method studies that did not separate qualitative findings and quantitative data separately were also excluded. Additional exclusion criteria included studies published only in abstract forms and those published in languages other than English (Supplementary S2).

### 2.3. Study Selection and Data Extraction

Titles and abstracts were independently screened by two reviewers on Covidence (EOK, KP, RDI, CA, AM, KM). Full-text articles that passed this stage were assessed for eligibility by a minimum of two authors (EOK, KP, RDI, CA, AM, KM). Any conflict in the screening process between reviewers was discussed with the senior reviewer (EYHT). For each study that met the inclusion criteria, key information was collected and standardised by assessing the study’s population, setting, methods and findings related to medication adherence in a pre-specified proforma.

### 2.4. Data Synthesis

Thematic analysis was carried out using the method described by Thomas and Harden [12] to assess and incorporate the qualitative data collected. Data analysis was conducted by 2 researchers independently (EOK and RDI). The extracted data was then organised from first order themes (participant quotes) and second order themes (author interpretations). Third order themes were then created through the synthesis and integration of the first- and second-order themes, forming a deeper understanding of factors influencing MA in post-stroke and TIA patients. The meta-synthesis was guided by five key research questions. These questions guided our data collection and analysis, providing insights into the challenges, supporting factors and real-life situations that influence medication adherence:

1. What are the views and experiences of those post-stroke or TIA in association with medication adherence?
2. What views do post-stroke or TIA patients have in relation to their condition and follow-up care?
3. What perspectives do post-stroke or TIA patients have in relation to medication taking?
4. What, if any, are the reasons or poor medication adherence in post-stroke or TIA patients?
5. What are some of the strategies employed to promote medication adherence reported by stroke and TIA patients?

### 2.5. Quality Assessment

The quality of each included study was assessed using the Joanna Briggs Institute (JBI) Critical Appraisal Checklist for Qualitative Research, which involves the appraisal of each study according to 10 criteria [13]. This includes congruity between research methodology and philosophical perspective, research question, methods, representation and analysis of data, and interpretation of results. It also looks at researcher characteristics, whether participant voices are adequately represented, whether the research was ethical and if the conclusions flow from the data [13].

## 3. Results

**Figure 1:**
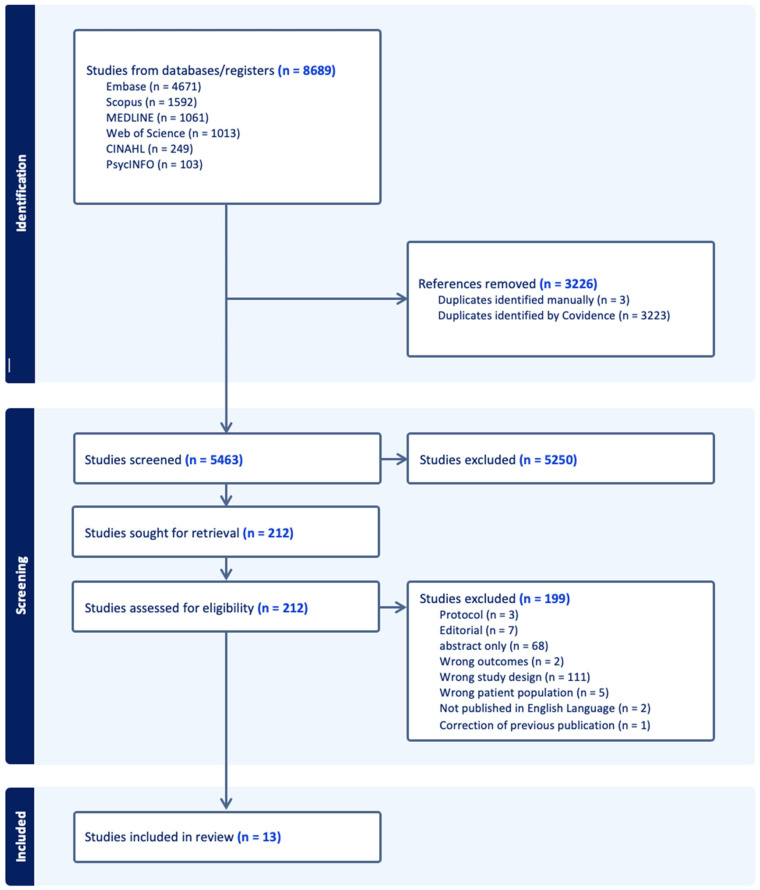
PRISMA flowchart.

In total 8689 papers were identified for review with a total of 5466 titles and abstracts screened by researchers (EOK, KP, RDI, CA, AM, KM), after 3223 were removed due to duplication. After title and abstract screening, 212 studies were identified as potentially relevant and underwent full text review and eligibility assessment by researchers (EOK, RDI, KP, CA, NW, AM) (see Figure 1).

### 3.1. Characteristics of Included Studies

Thirteen qualitative studies were included in this review from eight countries (United Kingdom *n* = 4 [14,15,16,17], Ireland *n* = 1 [18], USA *n* = 1 [19], France *n* = 1 [20], Germany *n* = 1 [21], China *n* = 3 [22,23,24], Malaysia *n* = 1 [25] and Australia *n* = 1 [26]) (see Table 1). A range of qualitative methods were used throughout the studies including 8 semi-structured or in-depth interviews [14,16,17,18,20,21,22,23,25], 4 focus groups [14,15,18,26] and 2 mixed method approaches, i.e., a qualitative study was embedded into other studies [19,24]. In the case of these two mixed method studies, both utilised in-depth interviews. Sample sizes per study varied from 8 to 126 participants, with a total of 435 across included studies. Participants included stroke and TIA survivors, family members of these patients, carers and healthcare professionals including nurses, pharmacists, speech and language therapists. Mean age of stroke and TIA participants ranged from 57 to 72 years. Some studies focused on the experiences of patients in relation to medication adherence post stroke/TIA, while others utilised the perspectives of healthcare professionals and explored factors impacting medication adherence in these patients.

### 3.2. Themes

**Figure 2:**
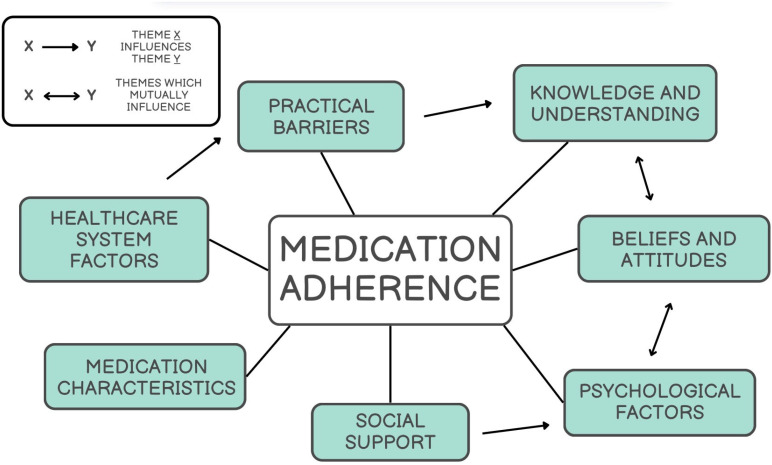
Linking of themes across studies.

Following data synthesis, seven overlapping themes emerged with evidence of mutual influence and connection to one another (see Figure 2). The themes are outlined in Supplementary S3 with exemplar first order quotes for each theme and subtheme provided.

#### 3.2.1. Knowledge and Understanding

This theme captures the extent to which stroke/TIA survivors demonstrated understanding of both the condition itself and the medications prescribed for secondary prevention. Levels of knowledge varied widely across studies. Some participants reported limited or delayed awareness of stroke, some only recognising its significance after experiencing the event themselves [17,20,25]. Survivors described difficulty in making sense of explanations by healthcare professionals, particularly when complex language was used, leaving them with only partial understandings of what they had experienced and what was needed of them to further prevent future ischaemic events. In contrast, others demonstrated a more comprehensive grasp of stroke, often linked to previous exposure or education [22]. For these individuals, knowledge about the importance of secondary prevention and risk factor management played a motivating role in adherence to medicine [19,25]. When it comes to understanding surrounding medications, this was inconsistent. Survivors frequently reported insufficient or unclear information about treatments, which contributed to confusion and poor adherence, even if it was unintentional [15,22,23]. Healthcare professionals also acknowledged that patients often failed to appreciate the longer-term goals which may be achievable with adherence, particularly when symptoms were absent [18,23]. At the same time, some participants described actively pursuing information about their medicines and developing a clearer sense of their role in secondary prevention, displaying the varied proactive nature between patients [15,23,25].

#### 3.2.2. Beliefs and Attitudes

The beliefs and attitudes of patients post-stroke/TIA have shown to be an influencing factor in medication adherence within this review. How the patient perceives their condition and the treatments associated weighs heavily in their likelihood to adhere to secondary preventative measures. For some participants, medicines were associated with reassurance and protection; they expressed confidence in their treatments, viewing them as essential in reducing the risk of further strokes [14]. Positive beliefs like this often manifested into patients being more willing to adhere, even if occasional forgetfulness occurred [24]. Some survivors emphasised the perceived importance of following medical advice and recognised adherence as a vital step in protecting their health [22,25]. In contrast, others showed scepticism, ambivalence, or outright resistance to treatment regimens [14,15,17]. Survivors sometimes questioned the effectiveness of prescribed treatments, framed medication as a burden, or minimised the value of lifestyle changes [14,15]. In certain cases, fatalistic attitudes, where patients attributed outcomes to fate or inevitability, efforts and motivation to adhere were lost [15,22]. HCPs also highlighted instances where patients showed indifference or reluctance, often describing these patients as passive in their involvement with their own care [18]. With patients who adopted these roles, necessity or lack of it was highlighted as a reasoning for these mentalities. Some survivors viewed adherence as critical, whereas others evaluated medicines in terms of risks vs. benefits, often relying heavily on trust in their doctors to navigate this balance, sometimes without even considering their own personal judgement [14,22,25].

#### 3.2.3. Practical Barriers

This theme captures the day-to-day obstacles that stroke and TIA survivors face in adhering to secondary prevention medications. Financial cost emerged as a recurrent issue across studies. Various included studies examined countries without universal healthcare coverage; patients disclosed the burden of ongoing prescription costs, with some opting for cheaper alternatives while recognising the need for adherence [18,22,26]. Cost also affected engagement with wider rehabilitation activities, such as exercise or physiotherapy programmes, which were viewed as highly valuable but unaffordable for those on low incomes [26]. Access to medication was another significant barrier, influenced both by physical limitations of patients post stroke/TIA and systemic healthcare factors [14,17]. Survivors with impairments, such as reduced mobility or one-sided weakness, found packaging difficult to open and tablets hard to manage alone [14]. Systemic challenges were particularly evident in rural or remote areas, where limited local services restricted access to rehabilitation and prescription renewals [26].

#### 3.2.4. Social Support

The role of social support networks emerged as central to how stroke and TIA survivors managed their medications post-discharge. Family and friends often played both a practical and emotional role, helping with organising and administering medicines and providing reminders when memory or motivation wavered. For some, family support helped to reduce the burden of management alone, while others described relatives adopting more of a caregiver role [16,21,22]. Although often beneficial, not all survivors welcomed this dynamic as some found family members to be overly persistent or “nagging,” preferring the more supportive influence of friends, although the former was more commonly seen within included studies [22]. HCPs were also seen as an important source of support. Effective communication about the purpose and importance of secondary prevention medication was expressed as motivating, with survivors placing strong trust in their clinicians. The multidisciplinary team (MDT) also played a role, with different professionals supporting both survivors and families in managing complex regimens and understanding the necessity of continued treatment [18,20]. Together, these findings highlight the necessity of social support in positively impacting medication adherence for this patient group.

#### 3.2.5. Healthcare System

Healthcare system factors played an important role in shaping how stroke and TIA survivors understood and engaged with their secondary prevention medications. One key issue was communication: many survivors noted difficulties in receiving clear and comprehensible information from doctors and pharmacists. Explanations were described as overly technical, rushed, or omitted altogether, leaving patients uncertain about the purpose of their medication and how best to manage it [20,22,25]. Alongside communication, issues from a systemic level were acknowledged. HCPs highlighted a lack of time and resources to provide comprehensive education and support, especially during hospital discharge or follow-up appointments [14,18]. Survivors in rural areas noted additional barriers in accessing re-examinations or ongoing support [23].

#### 3.2.6. Psychological Factors

Psychological wellbeing strongly influenced how stroke and TIA survivors engaged with their medicines and secondary prevention. Mental health difficulties, particularly depression and anxiety, were commonly described. These emotional responses decreased engagement among participants and, in some cases, led to total disconnection from actively participating in recovery. Some survivors felt a lack of purpose, while others identified clear goals, such as regaining independence or avoiding future strokes [14,18,25,26]. Within this review both adaptive and maladaptive coping mechanisms were disclosed by participants [21,22]. Positive coping included participation in rehabilitation therapies, adopting healthier lifestyles, or seeking social support, while negative coping included smoking, drinking alcohol, or abandoning efforts at risk factor management after setbacks as well as non-adherence to medications [18,25].

#### 3.2.7. Medication Characteristics

Side effects such as dizziness, fatigue, or disruptions to daily life were common concerns and, for some participants, trialling alternative therapies or skipping doses until symptoms improved were the preferred method of management here; seeking further assistance when side effects occurred was not mentioned [18,20,21]. Notably, many patients described feelings of worry around difficulties swallowing medication, despite most not having dysphagia. This fear concerned some participants and influenced their adherence to medications [18].

### 3.3. Risk of Bias

Quality appraisal according to the JBI Critical Appraisal Checklist for Qualitative Research generally showed strong standards across all included studies, scoring between a 7 and 9 out of 10 (see Supplementary S4). However, several recurring risks of bias were identified [5]. The criteria most frequently unmet were the absence of a clear statement acknowledging the researcher’s role, position or potential influence on data collection and interpretation, with this omission appearing in many studies [14,15,17,18,19,20,22,24,25]. In those scoring 9/10, there was a failure to report cultural or theoretical positioning, hindering insight into how backgrounds shaped analytic decisions [16,21,23,26]. A small number of studies showed gaps in reporting ethical approval or consent procedures [19,20]. One study demonstrated limited transparency regarding participation voice representation, with minimal participant quotes despite a larger sample [15].

## 4. Discussion

### 4.1. Summary of Findings

Secondary prevention following a stroke is delivered through defined clinical pathways, beginning with in-hospital initiation of medications including anti-hypertensives, lipid-lowering medication, and antiplatelet therapies. Medications are often lifelong to minimise the risk of recurrent stroke. Findings from this meta-synthesis highlight several opportunities for clinicians to strengthen support for MA among stroke and TIA survivors. Firstly, improving patient education is essential. Survivors frequently reported confusion about their condition and the purpose of secondary prevention medicines, suggesting that clinicians should prioritise jargon-free explanations and reinforce key messages at multiple points across the care pathway, as with any provision of information towards patients. This review also highlights the importance of personalised, patient-centred approaches. A previous systematic review in 2018 identified behavioural strategies as most effective in improving MA, with interventions delivered in healthcare settings more effective than those delivered in the home [27]. These findings were not specific to the stroke population, but may have relevance when considering how to implement our findings, with efficacy of each strategy needing to be weighed up against any associated cost. Both beliefs and emotional responses strongly shaped adherence behaviours and so clinicians should routinely explore patient perceptions and treatment concerns. Being mindful of the impact of mental health difficulties post-stroke will also be important in follow-up appointments as enabling early referral to psychological support services could also provide further holistic benefit including MA.

Themes drawn from the analysis demonstrate a wide variety of factors that may influence MA post-stroke, including behavioural, cognitive and individual health beliefs. Some of these factors are likely to be pre-existing prior to the stroke, whilst others, such as cognitive impairment, may occur as a result of the neurological injury caused by stroke. Risk factors may be modifiable or non-modifiable and this has implications for areas that future interventions may target. The weight of each individual factor is not currently known, and this would require further work to establish this, which is beyond the scope of this review.

### 4.2. Comparison with the Existing Literature

Stroke and TIA patients as well as HCPs included in this study agreed that there are difficulties in making sense of the information provided to them, which then contribute to confusion and non-adherence. These initial conversations and educational opportunities are key to long-term adherence. In a longitudinal survey of stroke patients, it was found that stroke patients do become non-adherent over time despite recognising the importance of these medications. Much of this was driven by what the patient’s beliefs were about the medications [28]. Interventions targeting these beliefs particularly around information about health consequences and social support can be used to modify medication-related beliefs to improve adherence [29]. This could be assisted through digital interventions such as remote consultations via telemedicine and mHealth interventions, which have been shown to be effective in improving MA in stroke patients [30]. Although these interventions can expand the potential of patient access to support from healthcare providers, not all stroke patients will have access to or be able to utilise such digital interventions particularly in resource-poor settings.

One of the challenges facing stroke patients is the fact that multimorbidity and polypharmacy (use of often five or more medications) are much more common in those with a diagnosis of stroke [31]. Although many will be needed to manage pre-existing co-morbidities alongside secondary prevention medication, there is also the possibility of inappropriate medication use in a large proportion of stroke patients [32]. The potential use of inappropriate medications, increasing complexity of medication regimes alongside the higher risk of side effects due to polypharmacy in stroke patients only adds to the challenges faced by stroke patients in MA. Although not specific to polypharmacy, participants in this study have also highlighted medication characteristics as a potential issue that HCPs need to help patients overcome. It is important to recognise that it may not always be clear who is able to provide this early and sustained intervention given that at the subacute stage patients are often being managed by hospital and community-based HCPs as well as by the primary care team. It may be that an HCP across the MDT is specifically tasked with this responsibility whilst other HCPs manage the consequences post-stroke. There are attempts at utilising the wider HCP community team including pharmacists. One example is the ADMED AVC study which looks to evaluate the efficacy of a pharmacist-led education programme to improve adherence to secondary prevention medication in stroke survivors [33]. Practice-based pharmacists have the appropriate expertise to not only assist with adherence but also to rationalise medication regimes to improve MA, with previous intervention trials showing that pharmacists are the most effective interventionists when looking to improve MA [27].

Stroke patients may experience complicated management plans necessitating personal investment. This additional work is known as treatment burden and is known to be associated with unfavourable outcomes [34]. As demonstrated by our evidence synthesis of reviews, social support is key to support the stroke-survivor which can in turn positively influence MA. In the context of polypharmacy, the patients’ social network can play a key role in MA [35]. Social networks have also been found to be a key factor in the stroke-survivors’ function with those with limited social support network having greater odds of functional decline [36]. Post-stroke isolation is also associated with more depressive symptoms in resource-poor settings [37]. It is evident therefore that the carers and family members of stroke-survivors are included in stroke discharge planning and their ongoing care not only to improve MA but also to maximise recovery and reduce the risk of some of the hidden effects such as post-stroke depression.

Many of the themes identified in this review align with those reported in the previous systematic review, including concerns about medication side effects, lack of understanding around medications, physical health issues, and the perceived benefit of the medication. New emerging themes include the impact of mental health on MA, which should prompt clinicians to consider this when discussing MA. Mental health may be negatively impacted by a range of challenges post-stroke, including occupational or financial strain, the emotional impact of physical limitations, and direct neurological injury [27].

### 4.3. Implications for Practice

Practical barriers identified display the need for proactive planning for these patients. Although the findings in this study may not be unique to stroke care and will undoubtedly exist in other chronic conditions, stroke patients do themselves present a unique challenge. Most post-stroke care focuses on physical rehabilitation. Whilst this is appropriate to maximise functional independence, there are often hidden impairments post-stroke that will affect MA. This includes, for example, cognitive impairment, depression and anxiety which can negatively influence patient behaviours not just in MA but in risk factor management as demonstrated by the participants included in our review. Primary care is generally where care takes place post-stroke. The multidisciplinary nature of primary care teams needs to be better utilised to ensure better medication adherence. For example, practice pharmacy teams could assist with home delivery services or assistive medication-taking devices where appropriate. They could also ensure MA through regular medication reviews. Social support emerged as a key facilitator of adherence, reinforcing how valuable the involvement of family members or carers during consultation and following treatment regimens can be. Some of this could be achieved using social prescriber link workers who can ensure stroke patients and their families are well supported in the community. Strengthening continuity of care, particularly in resource-limited areas, may further enhance long-term engagement in services. The General Practitioner is usually at the centre of long-term care and continuity has been found to reduce mortality [38]. However, it is important to recognise that not all strategies or interventions will be universal across all settings. For instance, social prescribers may not be accessible in all areas, and the availability of digital interventions may vary for patients. Clinicians need to balance the resources available with the individual needs of patients and should not assume that all interventions will work in every context.

Clinicians should also recognise the psychological impact of stroke when addressing MA. While our findings show the importance of managing mental health, particularly regarding the emotional response to physical limitations and cognitive impairment, addressing this should go hand in hand with strategies to help manage the practical aspects of medication taking. Early referral to psychological support services should be considered, but it should be acknowledged that access to these services may be limited, especially in resource-poor settings and alternative support strategies should be explored. Digital mental health interventions may be a valid option for some patients, but they should not replace in person care, particularly for those who struggle with technology which should be identified.

It is also essential to consider the complexity of medication regimens. Stroke patients often take multiple medications to manage both their stroke recovery and pre-existing comorbidities, which notably was a common theme in our synthesis for leading to confusion and/or nonadherence. Clinicians and pharmacists should provide appropriate medication reviews to reduce unnecessary medications and ensure that each prescribed medication has a clear purpose for the patient.

Ultimately, findings suggest that social support and patient-centred approaches are critical for improving MA, but clinicians must recognise that not all stroke survivors will have the same level of support, whilst cognitive impairments and individual beliefs may shape their ability to fully engage in education or interventions. Strategies should be tailored to individual patients’ needs, considering their cognitive, emotional and social challenges and it should be recognised that guidance regarding medication adherence should not be rigid, as each patient’s needs and circumstances will vary.

### 4.4. Strengths and Limitations

This systematic review has several strengths. We have been able to synthesise and harmonise data to provide an update on how clinicians and the healthcare system support stroke patients with MA. Further, our inclusive search has meant that we were able to identify literature from a wide range of settings across eight countries. We do recognise several limitations. We excluded studies that were not English language-based. The rationale behind this was due to the thematic data analysis that was completed to harmonise the results which relies on clarity and detail in the quotes provided by participants. This meant that non-English studies were excluded, which could have provided valuable insight into other healthcare systems. Further, many of the studies came from high income countries. Most of these countries will also have pre-specified follow-up post-stroke mechanisms and mature primary care settings which may minimise some of the barriers associated with MA post-stroke. We recognise that some of the challenges facing those from low-to-middle-income countries may have been missed particularly if their healthcare systems do not have clear ownership as to who manages those in their lives after stroke.

We recognise the inconsistent reporting of neurological impairment and disease severity across the included studies. While these factors are likely important in understanding the complex nature of MA, the lack of consistent data on stroke severity and neurological impairments prevents us from fully addressing how these factors might influence MA. This limits the clinical interpretation of our findings, as neurological factors (such as cognitive or functional impairments) could significantly alter adherence patterns. Future research should explicitly consider these variables to clarify their role in influencing MA post-stroke.

This systematic review did not incorporate quantitative data, which therefore limits the ability to evaluate the relevance of individual factors contributing to MA post-stroke. The rationale for exclusion of this data set was to prioritise depth of understanding whilst aiming to capture the complex, inter-dependent factors that can contribute to MA post-stroke. Future research using mixed-methods or quantitative analysis would be valuable to test the generalisability of these findings whilst also identifying the relative importance of individual risk factors for poor MA.

## 5. Conclusions

This updated meta-synthesis of qualitative studies has highlighted the increasing complexity that surrounds the stroke patient, their carers and the HCPs that care for them in relation to medication adherence. Person-centred holistic care is the key not just to improve MA but to improve the lives of those affected by stroke. Interventions designed to improve MA need to be person-centred but also to make use of the wider MDT that exists in the community to combat some of the challenges faced by stroke patients and their families.

## Figures and Tables

**Table 1:** Characteristics of included studies.

First Author & Year	Location	Participant Demographic	Time Since Event	Mean Age (Range)	Data Collection Method(s)	Aim(s) of Study
Bell, 2023 [18]	Ireland	14 participants (6 pharmacists, 4 speech and language therapists, 4 occupational therapists) 13 female and 1 male.	Not applicable All participants were HCPs.	Not stated	Semi-structured interviews and focus groups	Investigate the knowledge, attitudes, beliefs, and challenges faced by healthcare professionals in the continuity of care post-discharge from a hospital stroke ward, and its impact on medication adherence.
Gibson, 2021 [14]	United Kingdom	27 participants (9 stroke survivors, 3 informal carers, 15 nurses) Stroke survivors—6 female and 3 male. No comment made on sex of nurses or informal carers.	0–2 months	61.8 (52 to 76) within stroke survivor group	Semi-structured interviews—individual and small-group	Explore stroke survivors’, carers’ and nurses’ views and experiences about adhering to medication early after post-stroke hospital discharge.
Viprey, 2020 [20]	France	36 participants (22 stroke survivors, 14 TIA patients) 16 female and 20 male.	12 months	71.4 (38 to 97)	Exploratory and semi-structured interviews	Explore barriers and facilitators to adherence of secondary prevention medication after ischaemic stroke or TIA.
Lin, 2022 [22]	China	19 stroke survivors 8 female and 11 male.	Not stated	65.2 (42 to 89)	Semi-structured interviews	Understand how survivors of stroke perceive secondary prevention and explore their perceived barriers and facilitators using the Theoretical Domains Framework.
Xu, 2021 [23]	China	36 participants (14 healthcare providers, 10 stroke patients, 8 caregivers, 3 private sector employees, 1 government official)	Not stated	Not stated	Semi-structured interviews	Examine stakeholder perspectives on barriers to medication adherence in stroke patients, identifying opportunities to improve care and policy in resource-constrained settings.
Gong, 2019 [24]	China	8 stroke patients and caregivers. Breakdown not specified.	Not stated	Not stated	Mixed methods study, including literature review, expert consultation, qualitative in-depth interviews, and field-based pilot study followed by surveys and interviews.	To develop and pilot-test a mobile phone message-based package, as a component of a wider intervention to improve the health of stroke patients in resource-poor settings (SINEMA).
White, 2019 [19]	United States of America	43 participants (18 stroke survivors, 15 family members of stroke survivors, 3 nurses, 2 physicians, 1 social worker, 1 rehabilitation manager, 1 speech therapist, 1 psychologist, 1 physician assistant)	Within the last 12 months	57 (±8.7) for stroke survivors and 56 (±14.2) for family members	Mixed methods study, with a qualitative descriptive study and a quantitative cross-sectional study.	Describe control of risk factors after stroke from the perspectives of the stroke survivor, family, and healthcare professionals.
Appalasamy, 2019 [25]	Malaysia	10 stroke survivors 5 female and 5 male.	Within the last 6 months	57 (44–78)	In-depth interviews	Explore fundamental needs and barriers of medication taking self-efficacy in post-stroke patients.
Hewitt, 2024 [15]	United Kingdom	48 participants 16 stroke survivors 32 healthcare professionals 8 female and 8 male stroke survivors. 15 female and 17 male healthcare professionals.	Not Stated	64.3 (54–80) for stroke survivors 45.3 (32–60) for healthcare professionals	Focus group interviews	Explore factors influencing the prescribing and offering of diabetes treatments and the compliance with that treatment by stroke survivors.
Firth, 2023 [26]	Australia	20 participants (17 stroke survivors 3 spouses assisting with communication) 9 female and 11 male.	1–42 years	Not stated	Semi-structured focus groups and individual interviews	Investigate factors which influence stroke survivors’ decision-making about their rehabilitation and the prospect of taking recovery-promoting drugs.
Jamison, 2018 [16]	United Kingdom	126 participants 42 interview participants (28 stroke survivors 14 caregivers) 84 online forum participants (49 stroke survivors and 33 caregivers, 2 unknown) Of interview participants, 7 male survivors, 21 female survivors, 4 caregivers of male survivors and 10 caregivers of female survivors. Of online forum participants, 20 male survivors, 26 female survivors, 20 caregivers of male survivors and 12 caregivers of female survivors. 3 survivors of unknown gender and 3 participants of unknown gender and identity.	Within 5 years	72 (61–92) for interview participants	Semi-structured interviews and online forum	Determine the appropriateness of an online forum compared to face-to-face interviews as a source for qualitative research on adherence to secondary prevention medications after stroke.
Kindermann, 2023 [21]	Germany	17 TIA patients.	3 months	66 (56–76)	Semi-structured interviews	Identify common coping strategies and the possible occurrence of posttraumatic growth in TIA patients.
DaCosta, 2019 [17]	United Kingdom	31 participants (15 stroke survivors, 16 community pharmacists) 8 female and 7 male stroke survivors 8 female and 8 male community pharmacists.	First few months post-event, specific cut-off not stated	69.5 (48–91) for stroke survivors	Semi-structured interviews	Develop a tool to support medicine-focused person-centred consultations between community pharmacists and stroke survivors.

## Data Availability

No new data were created or analysed in this study.

## Supplementary Materials

The following supporting information can be downloaded at: https://www.mdpi.com/article/10.3390/neurolint18020034/s1, Supplementary S1: Search Strategy; Supplementary S2: Full text exclusions; Supplementary S3: Exemplar first order constructs (participant quotations) from included qualitative studies; Supplementary S4: Risk of Bias; Supplementary S5: PRISMA Checklist.

## Abbreviations

The following abbreviations are used in this manuscript:

HCP	Healthcare professional
JBI	Joanna Briggs Institute
MA	Medication adherence
TIA	Transient ischaemic attack
