# Supplementary material for: Medication Adherence Following Stroke and TIA: A Qualitative Synthesis of Patient, Caregiver and Clinician Perspectives
Source: Neurol Int. 2026 Feb 11;18(2):34. doi: 10.3390/neurolint18020034 (PMC12943394; doi:10.3390/neurolint18020034)
Supplement: Supplementary file 1 [file neurolint-18-00034-s001.zip › neurolint-4082725-supplementary.pdf]

Supplementary S1 - Search Strategy:

1 exp Stroke/
2 (stroke or ((cerebrovascular or brain or cerebral) and (arrest or failure or injury or insufficiency or insult)) or Transient Isch?emic Attack or TIA or Cerebrovascular Accident or CVA or brain vascular accident or cerebro?vascular accident).mp.
3 (medication or relapse prevention* or pharmacotherap* or Secondary Prevention).mp.
4 exp secondary prevention/ or exp drug therapy/
5 exp medication adherence/
6 (medication adherence or Compliance or Concordance or Persistence).mp
7 1 or 2
8 3 or 4
9 5 or 6
10 7 and 8 and 9
11 limit 10 to yr="2018 -Current"

Supplementary S2. Full text exclusions.

Author	Title	Year of publication	Reason for exclusion
Aakeroy R.; ; Gynnild M.N.; Lofblad L.; Dyrkorn R.; Ellekjaer H.; Lydersen S.; Helland A.; Spigset O.	Direct oral anticoagulant concentrations and adherence in stroke patients	2024	Wrong study design
Ahern E.; ; Dennehy K.; Gannon E.; Rubel M.H.; Ryan P.; Healy L	Statin prescribing in ischaemic stroke patients: Quality improvement of an acute stroke ward in a model 4 tertiary teaching hospital	2023	Abstract only
Aigbonoga, Daniel; ; Adewale, Boluwatife; Igwilo, Joshua; Adeyeye, Victoria; Olajide, Tobi; Olaniran, Oluwatosin; Akintayo, Abiodun; Aremu, Peter; Oluwadamilare, Faith; Popoola, Oluwafemi; Ogunniyi, Adesola	Efficacy of short message service (SMS) intervention on medication adherence and knowledge of stroke prevention among clinic attendees at risk of stroke: a randomized controlled trial.	2025	Wrong study design
Al Harbi A.	SECONDARY STROKE PREVENTION AMONG STROKE SURVIVORS IN RIYADH CITY, SAUDI ARABIA. RISKS, KNOWLEDGE, ADHERENCE TO STROKE DISCHARGE MEDICATIONS AND PREVENTIVE STRATEGIES	2023	Abstract only
Alhubaishi, Alaa; ; Almutairi, Maha A; Alasqah, Malak A; Alharthi, Shihanah H; Alqahtani, Abdulhadi M; Alnajjar, Lina I	Evaluation of Physicians' Compliance With Secondary Prevention Among Ischemic Stroke Patients: A Retrospective Study.	2023	Wrong study design
Alhusayni, S.F.; Abanmi, N.M.; Abanemai, N.A.; Alharbi, R.M.; Alatiyah, F.A.; Abdalla, S.M.; Alharbi, A.A. Neurosciences 2020;25(2):118-128	Secondary stroke prevention among stroke survivors in Riyadh City, Saudi Arabia	2020	Wrong study design

Al-Qahtani S.; ; Mason J.; Paudyal V.; Jalal Z	The role of pharmacists in providing pharmaceutical care in primary and secondary management of stroke: A systematic review	2020	Abstract only
Alqahtani S.; ; Paudyal V.; Wassel Y.; Mason J.; Jalal Z.	Adherence to secondary prevention medications in patients with stroke in Saudi Arabia	2021	Abstract only
Anderson C.S.; ; Chaturvedi S.	Stroke in China and India: Big populations, big challenges	2018	Abstract only
Anderson, Laura J; ; Nuckols, Teryl K; Coles, Courtney; Le, Michael M; Schnipper, Jeff L; Shane, Rita; Jackevicius, Cynthia; Lee, Joshua; Pevnick, Joshua M	A systematic overview of systematic reviews evaluating medication adherence interventions.	2020	Wrong study design
Ansari J.; ; Buchhanolla P.; Neshatfar A.; Parvathaneni A.; Triay R.; Gaudet A.; Abushehab N.; Cuellar-Saenz H.; Kelley R.; Javalkar V.	The Incidence and Clinical Outcomes of Acute Ischemic Strokesin Patients with Atrial Fibrillation on long term Oral Anticoagulation at OLSU-S	2022	Abstract only
Appalasamy, Jamuna Rani; ; Joseph, Joyce Pauline; Seeta Ramaiah, Siva; Quek, Kia Fatt; Md Zain, Anuar Zaini; Tha, Kyi Kyi	An Intervention to Promote Medication Understanding and Use Self-Efficacy: Design of Video Narratives for Aging Patients at Risk of Recurrent Stroke.	2019	Abstract only
Appalasamy J.R.; ; Ramaiah S.S.; Quek K.F.; Md Zain A.Z.; Tha K.K.	Medication Understanding and Taking Self-Efficacy Theory-Based Interventions: A Systematic Review	2020	Wrong study design
Appalasamy, J.R.; Tha, K.K.; Quek, K.F.; Ramaiah, S.S.; Joseph, J.P.; Md Zain, A.Z.	The effectiveness of culturally tailored video narratives on medication understanding and use self-efficacy among stroke patients	2018	Wrong study design

Appalasamy J.; ; Seeta Ramaiah S.; Joseph J.P.; Tha K.K.; Quek K.F.; Md Zain A.Z.	Narratives as a technique to improve medication understanding and use self-efficacy in stroke: Content development using the modified delphi method	2018	Wrong study design
Appalasamy J.; ; Selvaraj A.; Seeta Ramaiah S.	The Non-Adherence Scope in Stroke: A Narrative Review	2022	Wrong study design
Arkan, Gulcihan; ; Sarigol Ordin, Yaprak; Ozturk, Vesile; Ala, Rahmi Tumay	Investigation of Medication Adherence and Factors Affecting It in Patients With Stroke.	2022	Wrong study design
Azarenko, VJ; Potabashniy, VA; Fesenko, V; Knjazjeva, O	Adherence to treatment of patients with past ischemic stroke	2018	Not published in English Language
Babu V.; ; Soman B.; Zechariyah F.; Sampath S.; Geethu S.; Sivalekshmi S.; Sylaja P.N.	DEVELOPMENT AND EFFECT OF A COORDINATED COMMUNITY HEALTH WORKER (CHW) INTERVENTION (C-CHW-I) MODEL FOR THE STROKE SURVIVORS IN KERALA, SOUTH INDIA	2023	Abstract only
Badila E.; ; Weiss E.; Mihalcea V.; Balahura A.; Bartos D.; Predescu C	Stroke recurrence and compliance to antihypertensive treatment	2018	Abstract only
Basheti I.A.; ; Ayasrah S.M.; Ahmad M.	Identifying treatment related problems and associated factors among hospitalized post-stroke patients through medication management review: A multi-center study	2019	Wrong study design
Basheti, Iman A; ; Ayasrah, Shahnaz M; Ahmad, Muayyad M; Abu-Snieneh, Hana M; Abuadas, Fuad H	Medications adherence and associated factors among patients with stroke in the Kingdom of Saudi Arabia.	2022	Wrong study design

Bawa, Danish; ; Darden, Douglas; Ahmed, Adnan; Garg, Jalaj; Karst, Edward; Kabra, Rajesh; Pothineni, Krishna; Gopinathannair, Rakesh; Mansour, Moussa; Winterfield, Jeffrey; Lakkireddy, Dhanunjaya	Lower-adherence direct oral anticoagulant use is associated with increased risk of thromboembolic events than warfarin.	2024	Wrong study design
Ben Nasr, Nesrine; ; Rouault, Anne; Cornillet, Nicolas; Bruandet, Marie; Beaussier, Helene; Tersen, Isabelle; Bezie, Yvonnick; Zuber, Mathieu	Evaluation of a hypertension-based patient education program in a stroke center.	2018	Wrong study design
Braune I.; ; Kudrin B.; Wysota C.; Humayun A.; Goldmann E.; Parikh N.S.; Appleton N.; Boden-Albala B.	Using qualitative analysis to explore the themes of follow-up calls to discharged stroke patients	2018	Abstract only
Bridgwood, Bernadeta; ; Lager, Kate E; Mistri, Amit K; Khunti, Kamlesh; Wilson, Andrew D; Modi, Priya	Interventions for improving modifiable risk factor control in the secondary prevention of stroke.	2018	Wrong study design
Bridgwood B.; ; Lager K.E.; Mistri A.K.; Khunti K.; Wilson A.D.; Modi P.	Interventions for improving modifiable risk factor control in the secondary prevention of stroke	2018	Wrong study design
Buchinsky K.; ; Pope L.; Noah P.; Hackett C.; Cerejo R.	Stroke nurse navigation-evolving care delivery to effectively meet the needs of stroke patients and families	2022	Abstract only
Burzhunova M.; ; Alieva M.; Geraskina L.; Garabova N.; Fonyakin A.	Gender differences in patients with stroke and atrial fibrillation	2018	Abstract only
Cadel, Lauren; ; Cimino, Stephanie R; Bradley-Ridout, Glyneva; Hitzig, Sander L; Patel, Tejal; Ho, Chester H; Packer, Tanya L; Lofters, Aisha K; Hahn-Goldberg, Shoshana; McCarthy, Lisa M; Guilcher, Sara J T	Medication self-management interventions for persons with stroke: A scoping review.	2023	Wrong study design
Cao, Wenjing; ; Kadir, Azidah Abdul; Wang, Juan; Hu, Lin; Wen, Linlan; Yu, Mei; Peng, Liqun; Chen, Lanying; Luo, Na; Hassan, Intan Idiana	Medication non-adherence and associated factors among older adult stroke survivors in China.	2022	Wrong study design

Cao, Wenjing; ; Wang, Juan; Wang, Yuhui; Hassan, Intan Idiana; Kadir, Azidah Abdul	mHealth App to improve medication adherence among older adult stroke survivors: Development and usability study.	2024	Wrong study design
Cassidy R.; ; Dodds L.; Wilson P.; Hedayioglu J.; Hamilton-West K.; Corlett S	Why are younger stroke survivors poorer adherers?-qualitative evidence from the kemist study	2018	Abstract only
Cecchella, Ester; ; Bragazzi, Nicola Luigi; Cotellessa, Filippo; Campanella, William; Puce, Luca; Marinelli, Lucio; Curra, Antonio; Schenone, Cristina; Mori, Laura; Trompetto, Carlo	Barriers to Long-Term Adherence in Botulinum Toxin Therapy for Post-Stroke Spasticity: Insights and Implications from a Single-Center Study in North Italy.	2025	Wrong study design
Cengiz, Kayhan Nuri; ; Midi, Ipek; Sancar, Mesut	The effect of clinical pharmacist-led pharmaceutical care services on medication adherence, clinical outcomes and quality of life in patients with stroke: a randomised controlled trial.	2025	Wrong study design
Cheiloudaki, Emmanouela; ; Alexopoulos, Evangelos C	Adherence to Treatment in Stroke Patients.	2019	Wrong study design
Chen, Chen	Sex differences in poststroke outcomes among middle aged and older stroke survivors.	2025	Wrong study design
Chen, Chen; ; Reeves, Mathew J; Farris, Karen B; Morgenstern, Lewis B; Lisabeth, Lynda D	Sex Differences in Nonadherence to Secondary Stroke Prevention Medications Among Patients With First-Ever Ischemic Stroke.	2024	Wrong study design
Chen, Min-Jie; ; Wu, Chan-Chan; Wan, Li-Hong; Zou, Guan-Yang; Neidlinger, Susan Holli	Association Between Medication Adherence and Admission Blood Pressure Among Patients With Ischemic Stroke.	2019	Wrong study design

Chen, Yun; ; Zhang, Yuan; Jiang, Lianyan; Lu, Yanbin; Ding, Xiaojie; Jin, Wei; Xiong, Canxin; Huang, Daping	Investigation of vascular risk factor control and secondary prevention medication compliance in acute ischemic stroke.	2024	Wrong study design
Cholisoh Z.; ; Karuniawati H.; Abdullah M.A.A.; Kormin F.B.	Predictors of medication adherence behavior to secondary stroke prevention therapy among stroke survivors	2019	Abstract only
Chung, Pil-Wook; ; Yoon, Byung-Woo; Lee, Yeong-Bae; Shin, Byoung-Soo; Kim, Hahn Young; Park, Jae Hyeon; Kim, Byung-Kun; Yoo, Bong-Goo; Shin, Won-Chul; Kim, Eung-Gyu; Do, Jin Kuk; Park, Kyung-Pil; Jung, Yohan; Seo, Woo-Keun; Han, Moon-Ku; Kim, Jei; Kim, Yongduk; Bang, Oh Young; Hwang, Yang-Ha; Cha, Jin-Hye; Kim, Young-Joo	Medication Adherence of Statin Users after Acute Ischemic Stroke.	2018	Wrong study design
Colangelo, G; Cano, D; Marichal, S; Baladas, M; sanchez, E; Paredes, CK; Guirao, C; Silva, Y; Ustrell, X; Purroy, F; Freitas, J; Pagola, J; Muchada, M; Rodriguez-Luna, D; Villatoro, NR; Garcia-Camba, AGT; Olive-Gadea, M; Rizzo, F; Gisbert, MR; Simonetti, R; Molina, C; Ribo, M; Rubiera, M	PREDICT-POOR_COMP: An artificial intelligence-based tool to predict poor medication compliance after stroke	2025	Abstract only
Colelli D.R.; ; Kamra M.; Rajendram P.; Murray B.J.; Boulos M.I.	Predictors of CPAP adherence following stroke and transient ischemic attack	2020	Wrong study design
Coombes, Judith A; ; Rowett, Debra; Whitty, Jennifer A; Cottrell, Neil W	Using a structured, patient-centred, educational exchange to facilitate a shared conversation about stroke prevention medications.	2020	Wrong study design
Dalli, Lachlan L; ; Andrew, Nadine E; Kim, Joosup; Cadilhac, Dominique A; Sanfilippo, Frank M; Thrift, Amanda G; Nelson, Mark R; Lannin, Natasha A; Olaiya, Muideen T; Ryan, Olivia F; Booth, Brenda; Gall, Seana; Kilkenny, Monique F	Understanding of medications and associations with adherence, unmet needs, and perceived control of risk factors at two years post-stroke	2022	Wrong study design
Dalli, Lachlan L; ; Kim, Joosup; Cadilhac, Dominique A; Greenland, Melanie; Sanfilippo, Frank M; Andrew, Nadine E; Thrift, Amanda G; Grimley, Rohan; Lindley, Richard I; Sundararajan, Vijaya; Crompton, Douglas E; Lannin, Natasha A; Anderson, Craig S; Whiley, Leanne; Kilkenny, Monique F	Greater Adherence to Secondary Prevention Medications Improves Survival After Stroke or Transient Ischemic Attack: A Linked Registry Study	2021	Wrong study design

Dalli, Lachlan L; ; Olaiya, Muideen T; Kim, Joosup; Andrew, Nadine E; Cadilhac, Dominique A; Ung, David; Lindley, Richard I; Sanfilippo, Frank M; Thrift, Amanda G; Nelson, Mark R; Gall, Seana L; Kilkenny, Monique F	Antihypertensive Medication Adherence and the Risk of Vascular Events and Falls After Stroke: A Real-World Effectiveness Study Using Linked Registry Data.	2023	Wrong study design
Dalli L.; ; Andrew N.; Kim J.; Cadilhac D.; Sanfilippo F.; Thrift A.; Nelson M.; Olaiya M.; Ryan O.; Gall S.; Kilkenny M.	Patient understanding of medications is associated with improved medication adherence and risk factor control after stroke	2021	Abstract only
Dalli, L; Andrew, N; Kim, J; Cadilhac, D; Sanfilippo, F; Thrift, A; Nelson, M; Olaiya, M; Ryan, O; Gall, S; Kilkenny, M	Topic: AS23 Stroke Prevention (primary and secondary) PATIENT UNDERSTANDING OF MEDICATIONS IS ASSOCIATED WITH IMPROVED MEDICATION ADHERENCE AND RISK FACTOR CONTROL AFTER STROKE	2021	Abstract only
D'Alton M.; ; Baby M.; Donaghy L.; Hamad M.; Dolan E.; Sheehan O.	Adherence to Secondary Preventative Medications One Year Post Stroke	2024	Abstract only
Deen, Tonya; ; Terna, Theresa; Kim, Elizabeth; Leahy, Brian; Fedder, Wende	The Impact of Stroke Nurse Navigation on Patient Compliance Postdischarge.	2018	Wrong study design
Della Vecchia C.; ; Preau M.; Carpentier C.; Viprey M.; Merson F.; Haesebaert J.; Termoz A.; Dima A.; Schott A.M.	"It hits us over the head you don't know, you don't understand": The role of patients' illness representations in improving health post-stroke outcomes	2018	Abstract only
Dennis M.; ; Forbes J.; Graham C.; Hackett M.; Hankey G.J.; House A.; Lewis S.; Lundstrom E.; Sandercock P.; Mead G.	Fluoxetine to improve functional outcomes in patients after acute stroke: The focus rct	2020	Wrong study design
Devyani, Azad; ; Banandur, Pradeep S; Sukumar, Gautham Melur; Kulkarni, Girish Baburao; Mythirayee, S; Rathore, Himani	Assessment of Stroke Case-fatality, Disability, Perceived Needs and Barriers for Care among	2025	Wrong study design

	First-ever Stroke Patients Attending a Tertiary Care Neuro-specialty Center in India: A Cross-sectional Study.		
Ding X.; ; Wing J.J.; Gibbs B.H.; Drum E.; Gutierrez D.; Albala B.; Boden-Albala B.	Social Activities Are Positively Associated With Medication Adherence In Stroke Survivors	2023	Abstract only
Dirickson, Amanda	Relationship Between Anticoagulant Medication Adherence and Satisfaction in Patients With Stroke.	2019	Editorial
Donneyong M.M.; ; Fischer M.A.; Langston M.A.; Joseph J.J.; Juarez P.D.; Zhang P.; Kline D.M.	Examining the drivers of racial/ethnic disparities in non-adherence to antihypertensive medications and mortality due to heart disease and stroke: A county-level analysis	2021	Wrong study design
Duenas M.E.; ; Navarro J.C.	Utilization of morisky medication adherence scale in patients with ischemic stroke recurrence	2018	Abstract only
Elhfnawy A.M.; ; Bieber M.; Schliesser M.; Kraft P.	Atypical presentation of giant cell arteritis in a patient with vertebrobasilar stroke: A case report	2019	Wrong study design
Espinoza M.D.Z.; ; Madsen T.E.	Nonadherence to Preventive Medications After Ischemic Stroke: Opportunities to Target Interventions by Sex, Race, and Ethnicity	2024	Wrong study design
Fan, Qianqian; ; Doshi, Kinjal; Narasimhalu, Kaavya; Shankari, G; Wong, Pei Shieen; Tan, Il Fan; Ng, Szu Chyi; Goh, Si Ying; Woon, Fung Peng; De Silva, Deidre Anne	Impact of beliefs about medication on the relationship between trust in physician with medication adherence after stroke.	2022	Wrong study design

Flink, Maria; ; Lindblom, Sebastian; Tistad, Malin; Laska, Ann Charlotte; Bertilsson, Bo Christer; Warlinge, Carmen; Hasselstrom, Jan; Elf, Marie; von Koch, Lena; Ytterberg, Charlotte	Person-centred care transitions for people with stroke: study protocol for a feasibility evaluation of codesigned care transition support.	2021	Protocol
Frank T.; ; Neumann J.; Assmann A.; Schreiber S.; Haghikia A.; Barleben M.; Sailer M.; Goertler M.	Predictors for Adherence to Recommended Anticoagulation after Stroke Unit Discharge in Patients with Atrial Fibrillation	2024	Wrong study design
Gardener H.; ; Rundek T.; Lichtman J.; Leifheit E.; Wang K.; Asdaghi N.; Romano J.G.; Sacco R.L.	Adherence to Acute Care Measures Affects Mortality in Patients with Ischemic Stroke: The Florida Stroke Registry	2021	Wrong study design
George J.; ; Sreelekshmi R.S.; Rajendran A.; Sreedharan S.E.; Sylaja P.N.	Impact of COVID-19 pandemic on secondary stroke prevention	2021	Abstract only
Gibson, Josephine M E; ; Miller, Colette; Coupe, Jacqueline; Jones, Stephanie P	Medication-taking after stroke: a qualitative meta-synthesis of the perspectives of stroke survivors, informal carers and health professionals.	2020	Wrong study design
Gu H.-Q.; ; Yang X.; Wang C.-J.; Zhao X.-Q.; Wang Y.-L.; Liu L.-P.; Meng X.; Jiang Y.; Li H.; Liu C.; Xiong Y.-Y.; Fonarow G.C.; Wang D.; Xian Y.; Li Z.-X.; Wang Y.-J.	Assessment of Trends in Guideline-Based Oral Anticoagulant Prescription for Patients with Ischemic Stroke and Atrial Fibrillation in China	2021	Wrong study design
Gu, WB; Gong, EY; Yan, LJ	MEDICATION ADHERENCE AMONG STROKE SURVIVORS IN RURAL CHINA: A MIXED METHODS STUDY	2018	Abstract only
Gynnild, M N; ; Aakeroy, R; Spigset, O; Askim, T; Beyer, M K; Ihle-Hansen, H; Munthe-Kaas, R; Knapskog, A B; Lydersen, S; Naess, H; Rosstad, T G; Seljeseth, Y M; Thingstad, P; Saltvedt, I; Ellekjaer, H	Vascular risk factor control and adherence to secondary preventive medication after ischaemic stroke.	2021	Wrong study design

Gynnild M.N.; ; Aakeroy R.; Spigset O.; Ellekjaer H.	Adherence to secondary preventive anticoagulation and risk for subsequent stroke and death	2020	Abstract only
Gynnild M.; ; Rosstad T.; Naess H.; Ellekjaer H.	Adherence to secondary prevention after stroke-the nor-coast study	2018	Abstract only
Han Y.K.; ; Rajabalaya R.; Binti Pg Hj Mohammad Yassin D.H.N.; David S.R.	Medication adherence in stroke patients in Brunei Darussalam Public Hospital: the cross-sectional study associated with chronic diseases, life style and potential barriers	2020	Wrong study design
Han Y.; ; Wang M.; Wang C.; Li Z.; Wang Y.	SEX DIFFERENCES AND TIME TRENDS IN STROKE CARE AMONG ACUTE ISCHEMIC STROKE PATIENTS IN CHINA AND STROKE SERVICES	2023	Abstract only
Harbison, Joseph; ; McCormack, Joan; Brych, Olga; Collins, Ronan; O'Connell, Niamh; Kelly, Peter J; Cassidy, Tim	Anticoagulation usage and thrombolytic therapy in subjects with atrial fibrillation-associated ischemic stroke.	2025	Wrong study design
Hawking, Meredith K D; ; Robson, John; Taylor, Stephanie J C; Swinglehurst, Deborah	Adherence and the Moral Construction of the Self: A Narrative Analysis of Anticoagulant Medication.	2020	Wrong patient population
Hill, James; ; Harrison, Joanna; Raj, Sonia; Gregary, Bindu; Timoroksa, Anne-Marie; Gibson, Josephine	Mediators, confounders and effectiveness of interventions for medication adherence after stroke.	2020	Wrong study design
Hoarau, Damien; ; Ramos, Ines; Termoz, Anne; Fernandez, Violaine; Rambure, Marie; Allemann, Samuel S; Derex, Laurent; Haesebaert, Julie; Schott, Anne-Marie; Viprey, Marie	Determinants of adherence to post-stroke/transient ischemic attack secondary prevention medications: A cohort study.	2024	Wrong study design

Hsieh C.-Y.	Medication adherence and stroke prevention: What real world data tells us	2019	Editorial
Jamison, James; ; Ayerbe, Luis; Di Tanna, Gian Luca; Sutton, Stephen; Mant, Jonathan; De Simoni, Anna	Evaluating practical support stroke survivors get with medicines and unmet needs in primary care: a survey.	2018	Wrong study design
Jang, Dong Eun; ; Zuniga, Julie Ann	Factors associated with medication persistence among ischemic stroke patients: a systematic review.	2020	Wrong study design
Jenkins, Carolyn; ; Burkett, Nina-Sarena; Ovbiagele, Bruce; Mueller, Martina; Patel, Sachin; Brunner-Jackson, Brenda; Saulson, Raelle; Treiber, Frank	Erratum to stroke patients and their attitudes toward mHealth monitoring to support blood pressure control and medication adherence.	2019	Correction of previous publication
Jones S.; ; Levy S.; Johnson A.; Duncan P.; Cummings D.; Bushnell C.	Medication management related challenges identified at a poststroke/ transient ischemic attack (TIA) transitional care visit	2020	Abstract only
Kaddumukasa, Martin; ; Edwards, Alyssa M; Najjuma, Josephine Nambi; Mbalinda, Scovia Nalugo; Nakibuuka, Jane; Burant, Christopher J; Moore, Shirley M; Blixen, Carol; Katabira, Elly T; Sajatovic, Martha; Kaddumukasa, Mark	Evidence-Supported Interventions for Reducing Secondary Stroke Risk in Sub-Saharan Africa: A Systematic Review.	2023	Wrong study design
Kalkonde Y.; ; Abhishek S.	Mobile health for the secondary prevention of stroke	2023	Editorial
Kamal, Hazem; ; Khodery, Mohamed; Elnady, Hassan; Borai, Ahmed; Schaefer, Jan Hendrik; Fawi, Gharib; Steinmetz, Helmuth; Foerch, Christian; Spitzer, Daniel	Adherence to Antithrombotic Treatment and Ischemic Stroke Recurrence in Egypt and Germany: A Comparative Analysis.	2021	Wrong study design

Kamoen O.; ; Mahieu D.; Standaert D.; Derous J.; Lorrez A.; Beeckx F.; Maqueda V.; Yperzeele L.; Vanhooren G.; Vanacker P.	A personalized, digital coaching program after stroke (beroertecoach. be): Patients and caregivers view on usability and applicability	2018	Abstract only
Kernan W.N.; ; Viscoli C.; Young L.; Gorman M	Adherence and adherence recovery in a stroke trial	2019	Abstract only
Kern, Kyle C; ; Crossley, Alexander; Wu, Naomi; Mun, Katherine T; Dergalust, Sunita; Hinman, Jason D	Suboptimal medication possession ratio is associated with recurrent ischemic stroke in a veteran population.	2025	Wrong study design
Khan B.; ; Goldmann E.; Parikh N.; Appleton N.; Boden-Albala B.	Previous stroke/TIA history is associated with low medication adherence in a multi-ethnic cohort of stroke survivors	2018	Abstract only
Khan I.; ; Shechter A.; Diaz K.M.; Ammie J.; Chang B.; Edmondson D.; Kronish I.; Cruz G.; Murdock M.	POSTTRAUMATIC STRESS DISORDER AFTER SUSPECTED STROKE AND NON-ADHERENCE TO MEDICATION	2024	Editorial
Kilkenny M.; ; Dalli L.; Kim J.; Cadilhac D.A.; Sanfilippo F.; Olaiya M.; Thrift A.; Nelson M.; Ung D.; Sundararajan V.; Andrew N.	Utilisation of general practice management plans after stroke improves medication adherence: Linked data from a National Stroke Registry	2021	Abstract only
Kilkenny M.; ; Dalli L.; Olaiya M.; Cadilhac D.; Andrew N	ETHNIC DISPARITIES IN MEDICATION ADHERENCE AMONG PATIENTS WITH STROKE IN AUSTRALIA: A REAL-WORLD STUDY USING LINKED REGISTRY DATA	2022	Abstract only
Kim, Gye-Gyoung; ; Chae, Duck-Hee; Park, Man-Seok; Yoo, Sung-Hee	Factors Influencing 1-Year Medication Adherence of Korean Ischemic Stroke Survivors.	2020	Wrong study design
Kim, Gye-Gyoung; ; Chae, Duck-Hee; Park, Man-Seok; Yoo, Sung-Hee	Factors influencing 1-year medication adherence of Korean Ischemia stroke survivors.	2020	Wrong study design

Kim, Gye-Gyoung; ; Yoo, Sung-Hee; Park, Man-Seok; Park, Hyun-Young; Cha, Jae-Kwan	Factors Related to Beliefs about Medication in Ischemic Stroke Patients.	2022	Wrong study design
King A.; ; Bryan J.; Safford M.M.; Riffin C.; Adelman R.; Roth D.; Sterling M.R.	Caregiver strain is associated with medication adherence among caregivers with diabetes: The reasons for geographic and racial differences in stroke (REGARDS) study	2019	Abstract only
Kiran, Akshatha; ; Viscoli, Catherine M; Furie, Karen L; Gorman, Mark; Kernan, Walter N	Adherence to study drug in a stroke prevention trial	2020	Wrong study design
Kolmos, Mia; ; Christoffersen, Laura; Kruuse, Christina	Recurrent Ischemic Stroke - A Systematic Review and Meta-Analysis.	2021	Wrong study design
Kvarnström, K; Westerholm, A; Airaksinen, M; Liira, H	Factors Contributing to Medication Adherence in Patients with a Chronic Condition: A Scoping Review of Qualitative Research	2021	Wrong patient population
Kyrychenko O.	ANALYSIS OF COMPLIANCE WITH ANTICOAGULANT THERAPY IN PATIENTS WITH ISCHEMIC STROKE AND ATRIAL FIBRILLATION	2022	Abstract only
Lank, Rebecca J; ; Lisabeth, Lynda D; Levine, Deborah A; Zahuranec, Darin B; Kerber, Kevin A; Shafie-Khorassani, Fatema; Case, Erin; Zuniga, Belinda G; Cooper, George M; Brown, Devin L; Morgenstern, Lewis B	Ethnic Differences in 90-Day Poststroke Medication Adherence.	2019	Wrong study design
Lank R.; ; Lisabeth L.; Li C.; Kerber K.; Case E.; Garcia N.; Morgenstern L.B.	Ethnic differences in post-stroke medication adherence	2018	Abstract only

Levine, Deborah A; ; Burke, James F; Shannon, Colman F; Reale, Bailey K; Chen, Lena M	Association of Medication Nonadherence Among Adult Survivors of Stroke After Implementation of the US Affordable Care Act.	2018	Wrong study design
Li, Dong-Mei; ; Lu, Xiao-Ying; Yang, Peng-Fei; Zheng, Jing; Hu, Huan-Huan; Zhou, Yu; Zhang, Ling-Juan; Liu, Jian-Min	Coordinated Patient Care via Mobile Phone-Based Telemedicine in Secondary Stroke Prevention: A Propensity Score-Matched Cohort Study.	2023	Wrong study design
Liljehult J.; ; Molsted S.; Moller T.; Overgaard D.; Adamsen L.; Jarden M.; Christensen T.	Lifestyle counselling as secondary prevention in patients with minor stroke and transient ischemic attack: Study protocol for a randomized controlled pilot study	2020	Protocol
Lin, Chung-Ying; ; Ou, Huang-Tz; Nikoobakht, Mehdi; Brostrom, Anders; Arestedt, Kristofer; Pakpour, Amir H	Validation of the 5-Item Medication Adherence Report Scale in Older Stroke Patients in Iran.	2018	Wrong study design
Lindblom, Sebastian; ; Flink, Maria; von Koch, Lena; Tistad, Malin; Stenberg, Una; Elf, Marie; Carlsson, Axel C; Laska, Ann Charlotte; Ytterberg, Charlotte	A person-centred care transition support for people with stroke/TIA: A study protocol for effect and process evaluation using a non-randomised controlled design.	2024	Protocol
Lindblom S.; ; Ytterberg C.; Flink M.; Carlsson A.; Stenberg U.; Tistad M.; Von Koch L.; Laska A.C.	A feasibility study of an intervention to enhance self-management of prescribed medication for stroke secondary prevention	2023	Abstract only
Liu, YJ; Wei, M; Guo, LN; Guo, YL; Zhu, YR; He, Y	Association between illness perception and health behaviour among stroke patients: The mediation effect of coping style	2021	Wrong study design
Liyanage-Don, Nadia; ; Birk, Jeffrey; Cornelius, Talea; Sanchez, Gabriel; Moise, Nathalie; Edmondson, Donald; Kronish, Ian	Medications as Traumatic Reminders in Patients With Stroke/Transient Ischemic Attack-Induced Posttraumatic Stress Disorder.	2021	Wrong study design

Lowres N.; ; Giskes K.; Freedman B.	Next frontier for stroke prevention in atrial fibrillation: Ensuring anticoagulant persistence	2021	Editorial
Luthra, S; Crossley, A; Butani, PB; Suh, HC; Kamali-Grigorian, M; Dergalust, S; Liu, J; Wu, NM; Rao, NM; Wallis, RA; Hinman, JD	Medication Adherence as a Modulator of Ischemic Stroke Recurrence in the Veteran Population - The MISTER-VA Study	2020	Abstract only
Manohar Babu S.; ; Aravinda Swami P.; Kilari V.K.; Kakumani M.; Vasantha S.P.	Assessment of drug related problems in stroke patients	2019	Wrong study design
Mansoor, Hend; ; Manion, Daniel; Kucharska-Newton, Anna; Delcher, Chris; Lo-Ciganic, Wei-Hsuan; Jicha, Gregory A; Moga, Daniela C	Sex Differences in Prescription Patterns and Medication Adherence to Guideline-Directed Medical Therapy Among Patients With Ischemic Stroke.	2025	Wrong study design
Masjuan, J; ; Gallego, J; Aguilera, J M; Arenillas, J F; Castellanos, M; Diaz, F; Portilla, J C; Purroy, F	Use of cardiovascular polypills for the secondary prevention of cerebrovascular disease.	2021	Not pubished in English Language
Mathew D.T.; ; John S.K.; Souza D.D.; Sarma G.; Nadig R.; Badachi S.; Awati A.	Hurdles in adherence to antiplatelet drugs in patients with recurrent strokes	2019	Abstract only
Mavrokefalou E.; ; Tomazou G.; Ntavidi S.; Chioti A.; Kylla M.; Polymniou V.; Kamarinopoulos D.; Marakomichelakis G.	Achievement of therapeutic objectives of secondary patient prevention with acute ischemic cerebrovascular stroke	2022	Abstract only
Miethe J.; ; Franzisket C.; Wagner M.; Galle G	Cross-sector care process after stroke	2021	Abstract only
Mockler P.; ; Walmsley N.	Do health professionals assess stroke survivors' ability to self medicate on an acute stroke unit? an audit of current practice	2019	Abstract only

Mondesir, Favel L	Exploring the relationship between social support, pharmacy access and medication adherence.	2019	Abstract only
Murad, Hussam; ; Basheikh, Mohammed; Zayed, Mohamed; Albeladi, Roaa; Alsayed, Yousef	The Association Between Medication Non-Adherence and Early and Late Readmission Rates for Patients with Acute Coronary Syndrome.	2022	Wrong patient population
Myers J.; ; Bravata D.M.; Sico J.; Myers L.; Chaturvedi S.; Cheng E.; Baye F.; Zillich A.J.	The quality of medication optimization among patients with transient ischemic attack or minor stroke	2020	Wrong study design
Nakka V.; ; Pusapati S.; Manukonda A.; Kumaravelu S.; Saka V.P.	Assessment of drug compliance and targeted pharmacist intervention in acute ischemic stroke patient	2021	Abstract only
Neil W.P.; ; Burchette R.J.; Shiokari C.E.; Ovbiagele B	Are depressed stroke survivors less likely to be adherent to secondary prevention therapies?	2019	Abstract only
Ojo, Temitope; ; Ryan, Nessa; Birkemeier, Joel; Appleton, Noa; Ampomah, Isaac; Glozah, Franklin; Adongo, Philip Baba; Adanu, Richard; Boden-Albala, Bernadette	Adapting a skills-based stroke prevention intervention for communities in Ghana: a qualitative study.	2020	Wrong patient population
Ooi S.L.; ; Tan K.M.; Chee S.H.; Wong K.Y.	Adherence and persistence in NOAC treatment in older persons with atrial fibrillation and ischaemic stroke	2024	Abstract only
Patorno, E; Schneeweiss, S; Pawar, A; Mogun, H; Schwamm, L	Linking the Coverdell Clinical Stroke Program Inpatient Registry to Commercial Claims Data to Assess Post-Discharge Medication Adherence	2020	Abstract only

Pedersen R.A.; ; Petursson H.; Hetlevik I.	Stroke follow-up in primary care: a prospective cohort study on guideline adherence	2018	Wrong study design
Pishkhani, MK; Dalvandi, PA; Ebadi, A; Hosseini, MA	Adherence to a Rehabilitation Regimen in Stroke Patients: A Concept Analysis	2020	Wrong study design
Quek Y.P.; ; Lim P.K.; Tan I.F.; Wai May N.; De Silva D.	ISSUES DETECTED THROUGH PERIODIC OPPORTUNISTIC SCREENING BY A STROKE LIAISON OFFICER (SLO) IN THE FIRST YEAR AFTER STROKE ADMISSION	2022	Abstract only
Rattanayotin, J; Vanijja, V	Designing and Developing Android Application for Medication Reminder to Improve Treatment Efficiency of Stroke Patient	2018	Wrong study design
Ray, Adrija; ; Ray, Tapobrata Guha; Pal, Jyotirmoy; Ray, Biman Kanti; Sanyal, Debasish; Dubey, Souvik	Adherence to anti-hypertensive medications and its determinants: A study among hypertensive stroke patients in a tertiary care government hospital of West Bengal.	2021	Wrong study design
Reyes R.; ; Coradin D.; Reyes Fernandez B.; Ouslander J.G.; Engstrom G.	Adherence to 2013 AHA/ACC treatment guidelines for secondary prevention after stroke or TIA in patients 75 years and older	2020	Abstract only
Robertson B.; ; Steiner N.; Wolber N.; Izzo R.; Castro M.; Figueroa S.; Paletz L.B.	Closing The Revolving Door: Reducing Stroke Readmissions	2023	Abstract only
Rohde, Daniela; ; Gaynor, Eva; Large, Margaret; Mellon, Lisa; Bennett, Kathleen; Williams, David J; Brewer, Linda; Hall, Patricia; Callaly, Elizabeth; Dolan, Eamon; Hickey, Anne	Cognitive impairment and medication adherence post-stroke: A five-year follow-up of the ASPIRE-S cohort.	2019	Wrong study design

Rowat, Anne	Commentary: Medication adherence early after stroke: using the Perceptions and Practicalities Framework to explore stroke survivors', informal carers' and nurses' experiences of barriers and solutions.	2021	Editorial
Ruksakulpiwat, Suebsarn; ; Benjasirisan, Chitchanok; Ding, Kedong; Phianhasin, Lalipat; Thorngthip, Sutthinee; Ajibade, Anuoluwapo D; Thampakkul, Jai; Zhang, Amy Y; Voss, Joachim G	Utilizing Social Determinants of Health Model to Understand Barriers to Medication Adherence in Patients with Ischemic Stroke: A Systematic Review.	2023	Wrong study design
Ruksakulpiwat, Suebsarn; ; Liu, Zhaojun; Yue, Shihong; Fan, Yuying	The Association Among Medication Beliefs, Perception of Illness and Medication Adherence in Ischemic Stroke Patients: A Cross-Sectional Study in China.	2020	Wrong study design
Saade S.; ; Kobeissy R.; Sandakli S.; Malaeb D.; Lahoud N.; Hallit S.; Hosseini H.; Salameh P.	Medication adherence for secondary stroke prevention and its barriers among lebanese survivors: A cross-sectional study	2021	Wrong study design
Sajatovic, Martha; ; Tatsuoka, Curtis; Welter, Elisabeth; Colon-Zimmermann, Kari; Blixen, Carol; Perzynski, Adam T; Amato, Shelly; Cage, Jamie; Sams, Johnny; Moore, Shirley M; Pundik, Svetlana; Sundararajan, Sophia; Modlin, Charles; Sila, Cathy	A Targeted Self-Management Approach for Reducing Stroke Risk Factors in African American Men Who Have Had a Stroke or Transient Ischemic Attack.	2018	Wrong study design
Sakr, Fouad; ; Dabbous, Mariam; Akel, Marwan; Salameh, Pascale; Hosseini, Hassan	Adherence to Post-Stroke Pharmacotherapy: Scale Validation and Correlates among a Sample of Stroke Survivors.	2022	Wrong study design
Sato, So; ; Sasabuchi, Yusuke; Okada, Akira; Yasunaga, Hideo	Do Orally Disintegrating Tablets Facilitate Medical Adherence and Clinical Outcomes in Patients with Post-stroke Dysphagia?.	2025	Wrong study design

Schwartz, JK; Aylmer, K; Tayeb, S; Hamdan, S	Comparisons of Medication Adherence in Adult Stroke & Hypertension: Implications for Applying the Health Self-Management Evidence in Disability	2024	Abstract only
Serber, Stacy L; ; Wachtel, Noah; Fox, Madison; Petrushonis, Corrine	A Multidisciplinary Approach to Increase Dysphagia Compliance in Stroke Patients.	2024	Wrong outcomes
Seregni A.; ; Tropea P.; Re R.; Biscaro V.; Judica E.; Caprino M.; Gand K.; Schlieter H.; Corbo M	Secondary care for subjects with stroke: Compliance, usability and technological acceptance of the vCare platform solution	2024	Wrong study design
Shani, SD; Kutty, VR; Varma, RP; Jissa, VT; Sylaja, PN	Facilitators and Barriers to Medication Adherence Among Stroke Survivors in India	2020	Abstract only
Shani, S D; ; Sylaja, P N; Sankara Sarma, P; Raman Kutty, V	Facilitators and barriers to medication adherence among stroke survivors in India.	2021	Wrong study design
Shani, S D; ; Varma, Ravi Prasad; Sarma, Sankara P; Sreelakshmi, R S; Harikrishnan, Ramachandran; Kutty, V Raman; Sylaja, P N	Treatment in a Stroke Unit and Risk Factor Control Reduce Recurrent Stroke Risk	2022	Wrong study design
Shani, SD; Varma, RP; Sarma, PS; Sylaja, PN; Kutty, VR	Life Style and Behavioural Factors are Associated with Stroke	2021	Wrong study design
Shani S.; ; Varma R.P.; Sarma P.S.; Sreelakshmi R.; Harikrishnan P.; Sylaja P.; Kutty V.R.	STROKE UNIT TREATMENT AND RISK FACTOR CONTROL REDUCE RECURRENT STROKE RISK	2022	Abstract only
Shankari G.; ; Ng R.S.C.; Goh S.Y.; Woon F.P.; Ganguly R.; Then L.Y.Y.; Doshi K.; Wong P.S.; De Silva D.A.	Adherence to secondary prevention medications in ischaemic stroke and TIA patients in Singapore	2019	Abstract only

Shankari, G; ; Ng, Szu Chyi; Goh, Si Ying; Woon, Fung Peng; Doshi, Kinjal; Wong, Pei Shieen; Fan, Qianqian; Tan, Il Fan; Narasimhalu, Kaavya; De Silva, Deidre Anne	Modifiable Factors Associated with Non-Adherence to Secondary Ischaemic Stroke Prevention Strategies.	2020	Wrong study design
Sheehan, Orla C; ; Dhamoon, Mandip S; Bettger, Janet Prvu; Huang, Jin; Liu, Chelsea; Rhodes, J David; Clay, Olivio J; Roth, David L	Racial differences in persistence to secondary prevention medication regimens after ischemic stroke.	2022	Wrong study design
Sjolander, Maria; ; Gustafsson, Maria; Holmberg, Henrik; Glader, E-L	Longitudinal changes in self-reported medication adherence and beliefs about post-stroke medicines in Sweden: a repeated cross-sectional study.	2024	Wrong study design
Skanes A.C.; ; Gula L.J.	Can We Anticipate Nonadherence to Anticoagulation?: Elegant Modeling, But No Clear Predictions	2021	Wrong study design
Sly D.; ; Husted M.; McKeague L.; Everington T.	"I just didn't want to trust it at all": Atrial fibrillation patient's treatment experience of rivaroxaban and warfarin	2022	Wrong patient population
Springer, Mellanie V; ; Skolarus, Lesli E; Patel, Minal	Food Insecurity and Perceived Financial Stress are Associated with Cost-related Medication Non-adherence in Stroke.	2023	Wrong study design
Srithumsuk, Werayuth; ; Chaleoykitti, Saisamorn; Jaipong, Saitip; Pattayakorn, Pinthusorn; Podimuang, Kattiya	Association between depression and medication adherence in stroke survivor older adults.	2021	Wrong study design
Sui, Weijing; ; Wan, Li-Hong	Association Between Patient Activation and Medication Adherence in Patients With Stroke: A Cross-Sectional Study.	2021	Wrong study design

Sylaja, P N; ; Babu, Veena; Sivasambath, S; Zechariah, Feba; Gireeshan, Sivalekshmi; Ganesan, Geethu; Soman, Biju; Singh, Gurpreet; Panniyamakal, Jeemon; Gopal, Bipin	Development and Effect of a Coordinated Community Health Worker Intervention Model for the Stroke Survivors in Kerala, South India.	2024	Wrong study design
Taylor T	Medication self-management for patients and families on an inpatient stroke rehabilitation unit	2019	Abstract only
Thompson A.; ; Dawson D.; Wang R.; Cameron J.; Nalder E.	EXPLORING PERSPECTIVES OF PEOPLE WITH STROKE, CAREGIVERS, AND HEALTH PROFESSIONALS ON MOBILE TECHNOLOGY FOR STROKE SELFMANAGEMENT SUPPORT: AN INTERPRETIVE DESCRIPTION STUDY	2023	Abstract only
Tian, Lulu; ; Wu, Juan; Qi, Zhan; Qian, Shijing; Zhang, Sha; Song, Danfei; Chen, Beilei; Zhu, Deqiu	Drug-related problems among community-dwelling elderly with ischemic stroke in China.	2023	Wrong study design
Tiu, Cristina; ; Grad, Diana Alecsandra; Muresanu, Dafin	Romanian registry for interventional treatment in acute stroke: a hope for secondary prevention through medication adherence improvement.	2023	Editorial
Turan, Tanya N; ; Al Kasab, Sami; Nizam, Azhar; Lynn, Michael J; Harrell, Jamie; Derdeyn, Colin P; Fiorella, David; Janis, L Scott; Lane, Bethany F; Montgomery, Jean; Chimowitz, Marc I	Relationship between Risk Factor Control and Compliance with a Lifestyle Modification Program in the Stenting Aggressive Medical Management for Prevention of Recurrent Stroke in Intracranial Stenosis Trial.	2018	Wrong study design
Turner G.M.; ; Calvert M.; Foy R.; Atkins L.; Collis P.; Tearne S.; Jowett S.; Handley K.; Mant J.	Structured follow-up pathway to address unmet needs after transient ischaemic attack and minor stroke (SUPPORT TIA): Feasibility study and process evaluation	2025	Wrong study design

van de Steeg, Bas J W; ; Esselink, Anne C; de Wit, Hugo A J M; Kramers, Cornelis; van den Bemt, Bart J F	Medication Adherence to Direct Oral Anticoagulants: Extent and Impact of Side Effects.	2024	Wrong study design
Varadan P.; ; Sivanandy P.; Hamzah N.	Stroke knowledge level among patients with ischemic stroke at Universiti Malaya Medical Centre	2024	Wrong study design
Vemuri, Ajith Kumar; ; Hejazian, Seyyed Sina; Sadr, Alireza Vafaei; Zhou, Shouhao; Decker, Keith; Hakun, Jonathan; Sciamanna, Christopher; Abedi, Vida; Zand, Ramin	Adherence to Physical Activity Recommendations Among Stroke Survivors in the United States.	2025	Wrong study design
Verma, SJ; S, ADK; Pandian, J	IDENTIFYING MEDICATION ADHERENCE CHALLENGES IN SECONDARY STROKE PREVENTION: DATA FROM THE SPRINT INDIA TRIAL	2024	Abstarct only
Viprey M.; ; Fokoun-Matchikou C.; Termoz A.; Haesebaert J.; Chakir M.; Della Vecchia C.; Tassa O.; Tazarourte K.; Nighoghossian N.; Cakmak S.; Meyran S.; Ducreux B.; Pidoux C.; Bony T.; Douplat M.; Potinet V.; Sigal A.; Derex L.; Schott A.M.	Factors influencing adherence to secondary prevention medication after ischemic stroke: A prospective population-based cohort study in RHONE area of france	2018	Abstract only
Wang, Jing; ; Zhao, Chen-Xi; Tian, Jin; Li, Yan-Ru; Ma, Kai-Fang; Du, Rui; Li, Meng-Kun; Hu, Rui	Effect of hospital-community-home collaborative health management on symptoms, cognition, anxiety, and depression in high-risk individuals for stroke.	2025	Wrong study design
Wangqin R.; ; Laskowitz D.T.; Li Z.; Wang Y.; Liu L.; Liang L.; Matsouaka R.A.; Saver J.L.; Fonarow G.C.; Bhatt D.L.; Smith E.E.; Schwamm L.H.; Bettger J.P.; Hernandez A.F.; Peterson E.D.; Xian Y.	International comparison of patient characteristics and quality of care for ischemic stroke: Analysis of the China National Stroke Registry and the American Heart Association get with the guidelines-Stroke program	2018	Wrong study design

Wang, Yilong; ; Li, Zixiao; Zhao, Xingquan; Liu, Liping; Wang, Chunxue; Wang, Chunjuan; Peterson, Eric D; Schwamm, Lee H; Fonarow, Gregg C; Smith, Sidney C Jr; Bettger, Janet; Wang, David; Li, Hao; Xian, Ying; Wang, Yongjun	Evidence-Based Performance Measures and Outcomes in Patients With Acute Ischemic Stroke.	2018	Wrong study design
Wang, YL; Li, ZX; Zhao, XQ; Liu, LP; Wang, CX; Wang, CJ; Peterson, ED; Schwamm, LH; Fonarow, GC; Smith, SC; Bettger, J; Wang, D; Li, H; Xian, Y; Wang, YJ	Evidence-Based Performance Measures and Outcomes in Patients With Acute Ischemic Stroke Findings From the China National Stroke Registry	2018	Wrong study design
Wawruch M.; ; Murin J.; Tesar T.	Non-persistence with statins in diabetic and non-diabetic patients with transient ischemic attack	2021	Abstract only
Wawruch M.; ; Wimmer G.; Murin J.	Risk factors for non-persistence with antiplatelet agents in patients after a transient ischemic attack	2019	Abstract only
Wawruch M.; ; Zatko D.; Wimmer G.; Luha J.; Murin J.; Tesar T.	Factors influencing non-persistence with antiplatelet medications and statins in patients after an ischemic stroke	2018	Abstract only
Wessol, Jennifer L; ; Russell, Cynthia L; Olds, Karin E	A Pilot Randomized Controlled Trial Testing the Feasibility and Acceptability of a SystemCHANGE Intervention to Improve Medication Adherence in Older Adult Stroke Survivors.	2019	Wrong study design
Widjaja, Karina Kumaladewi; ; Chulavatnatol, Suvatna; Suansanae, Thanarat; Wibowo, Yosi Irawati; Sani, Achmad Firdaus; Islamiyah, Wardah Rahmatul; Nathisuwan, Surakit	Knowledge of stroke and medication adherence among patients with recurrent stroke or transient ischemic attack in Indonesia: a multi-center, cross-sectional study.	2021	Wrong study design
Williams, Nadia A	Medication beliefs and behaviors of hypertensive Caribbean immigrants living in New York city.	2022	Abstract only

Wojcik R.; ; Greger J.; Zelen K.; Aladeen T.; Rainka M.; Westphal E.; Bates V.; Gengo F.	Risk of recurrent stroke or transient ischemic attack due to abrupt discontinuation of aspirin: A case series	2020	Abstract only
Wong, Pei Shieen; ; Narasimhalu, Kaavya; Tio, Siaw Li; Shankari, G; Doshi, Kinjal; De Silva, Deidre Anne	Adherence to secondary stroke prevention medications in Singapore: a single center study.	2024	Wrong study design
Wong P.S.; ; Narasimhalu K.; Tio S.L.; Shankari G.; Doshi K.; De Sila D.	MEDICATION CLASS AFFECTS COMPLIANCE IN ISCHEMIC STROKE PATIENTS	2023	Abstract only
Xiao, Yamei; ; Xu, Xiaomei	Enhanced stroke rehabilitation outcomes through Information-Motivation-Behavioral skills model and Hospital-Community-Family ternary linkage integration: A randomized controlled trial.	2025	Wrong study design
Xu J.; ; Prvu Bettger J.; Pan Y.; Liu L.; Li Z.; Wang Y.	Association of socioeconomic status with medication persistence and adherence after an acute ischemic stroke	2018	Abstract only
Yang J.; ; Yang M.; Cheng H.; Wang X.	Antithrombotics Prescription and Adherence Among Stroke Survivors: A Systematic Review and Meta-Analysis	2022	Abstract only
Yang, ZY; Chen, RX; Gao, LZ; Jin, LF	Medication adherence and health beliefs related to stroke prevention among older adult with hypertensive in China	2021	Abstract only
Yan, Xiuli; ; Liu, Zhuo; Guo, Zhen-Ni; Sun, Ye; Jin, Hang; Sun, Xin; Sun, Huijie; Yang, Yi	Positive Influence of Stroke Health Manager on Risk Factors Control and Medication Adherence After Ischemic Stroke.	2020	Wrong study design

Yap, Kwong Hsia; ; Warren, Narelle; Allotey, Pascale; Reidpath, Daniel D	Understandings of stroke in rural Malaysia: ethnographic insights.	2021	Wrong outcomes
Yeo, See-Hwee; ; Toh, Matthias Paul Han Sim; Lee, Sze Haur; Seet, Raymond Chee Seong; Wong, Lai Yin; Yau, Wai-Ping	Impact of medication nonadherence on stroke recurrence and mortality in patients after first-ever ischemic stroke: Insights from registry data in Singapore.	2020	Wrong study design
Yoo S.-H.; ; Kim K.-G.; Kim S.-R.; Park M.-S.; Kim J.-T.; Park H.-Y.; Yi S.-H.; Cha J.-K.; Kim D.-H.; Nah H.-W	3-month medication persistence and medication adherence in korean ischemic stroke survivors	2019	Abstract only
Yoo, Sung-Hee; ; Kim, Gye-Gyoung; Kim, Sung Reul; Park, Man-Seok; Kim, Joon-Tae; Choi, Kang-Ho; Park, Hyun-Young; Yi, Sang-Hak; Cha, Jae-Kwan; Kim, Dae-Hyun; Nah, Hyun-Wook	Predictors of long-term medication adherence in stroke survivors: A multicentre, prospective, longitudinal study.	2022	Wrong study design
Zhang Y.; ; Fan D	Assessment of smart phone wechat-based improvement services for ischemic stroke secondary prevention of discharge patients' treatment adherence: A cohortand case-control study	2020	Abstract only
Zheng S.; ; Lyu T.J.; Li Z.; Gu H.; Yang X.; Wang C.; Li H.; Jiang Y.; Shen H.; Wang Y.	GRP per capita and hospital characteristics associated with intravenous tissue plasminogen activator adherence rate: Evidence from the Chinese Stroke Center Alliance	2021	Wrong study design
Zhong, Jie; ; Gao, Yuguang; Huang, Deqing; Hu, Yueqiang; He, Qianchao; Diao, Limei; Hu, Yuying; Chen, Wei	Analysis of antiplatelet therapy adherence in patients with ischemic cerebral stroke.	2023	Wrong study design
Zhong, XM; Li, L; Ye, Q; Wang, J; He, LY; Li, CQ	Cognition and influencing factors of secondary prevention in patients with ischemic stroke 1 year	2025	Wrong study design

	after discharge in Southwest China: a cross-sectional survey		
Zhong, Xuemin; ; Li, Li; Ye, Qing; Wang, Jian; He, Lanying; Li, Changqing	Cognition and influencing factors of secondary prevention in patients with ischemic stroke 1 year after discharge in Southwest China: a cross-sectional survey.	2024	Wrong study design

Supplementary S3. Exemplar first order constructs (participant quotations) from included qualitative studies

Theme	Subtheme/Relevance of Quote	Participant quote	Participant	Study
**Knowledge and understanding**	Poor understanding of their condition	"They try to explain to me [what a stroke is], but it's hard to explain to people who aren't in the trade. And then, it's difficult to explain because, they use terms like… which aren't familiar to us."	Stroke survivor	Viprey 2020
		"I only learned more about stroke when I got one."	Stroke survivor	Appalasamy 2019
		"I didn't know it was a stroke until I woke up in… I knew after […] But I was still out of it so I didn't understand anything…"	Stroke survivor	Viprey 2020
	Good understanding of their condition	"I preferred reading books and watching TV, so I knew it was a stroke when it occurred, and I knew we should go to hospital as soon as possible. I also knew the importance of preventing it…"	Stroke survivor	Lin 2022
		"I knew I had high blood pressure but I wasn't really concerned about it so I didn't do the right things to control it. I thought I was too young to have a stroke but now I am motivated to make changes"	Stroke survivor	White 2019
		"Once you have a stroke, you need to read a lot to know more about it…we don't know when we can get it again."	Stroke survivor	Appalasamy 2019
	Poor understanding of medications	"Nobody told me clearly. They just provided similar, limited, even too-medical information, and I was confused about which instructions they provided were more suitable for me"	Stroke survivor	Lin 2022
		"Actually I think it's that their [patient's] understanding is insufficient, their understanding of the long- term treatment	HCP	Xu 2021

		goals is insufficient… They only see this time,"I'm good I don't have any symptoms. I'm good, so I will not take medicine or do follow-up examination."		
		"Yeah, I discovered I had type 2 diabetes as a result of my heart attack when they tested for it, and they said, ‘Oh, and you have got type 2 diabetes.' And it sorts of stopped at that, apart from they said, ‘Oh, here you are, take gliclazide because that helps.'"	Stroke survivor	Hewitt 2024
**Beliefs and attitudes**	Positive beliefs	"I feel okay ... the medication is doing what it is supposed to be doing ... I know that it thins the blood and ... it hopefully will prevent having another [stroke]"	Stroke survivor	Gibson 2021
		"If somebody said to me, take this pill and you will not have a stroke, I'd bite their arm off to go after that, and it's as simple as that because I'm looking round here and I'm one of the least affected by the stroke that I had..."	Stroke survivor	Hewitt 2024
		"It doesn't bother me to take it, and at a push I'd say, taking it reassures me. There you go, I tell myself that it reduces the risks and I don't want another [TIA] [. . .], it's really security for me."	Stroke survivor	Viprey 2020
	Negative beliefs	"sometimes they don't want to know ... sometimes they have no interest. They just are like I was told to take this so I'll take it ... It's scary how happy they are just to take what's prescribed sometimes"	HCP	Bell 2023
		"what's the point? Really, it's – you'll maybe get the balance. If I take this, that and this, what will I get away with? You know, how will I be able to cope, or if I do not take this, where am I heading? And it's just a question of balance and you are relying on the person that's talking to you, the doctor, to work out that balance, take this, take this, take this and this goes down. But other than that, you guess you'll be the perfect weight, the perfect, but you might have a stroke."	Stroke survivor	Hewitt 2024
		"... some people can be quite stubborn ... they'll say ‘Well ... I'm not taking it ... and that's it"	HCP	Gibson 2021

		“The medicine will definitely cause more side effect…it is toxic especially to your kidneys...you just need to relax to bring down the blood pressure, sometimes I control it myself”	Stroke survivor	Appalasamy 2019
	Perceived importance	“It is critical to adhere to physicians’ advice to take medication.”	Stroke survivor	Lin 2022
		“Indeed, I never forget to take drugs. It is important, I know”	Stroke survivor	Lin 2022
		“We need to take the medicine, if not it can worsen our condition; that’s what I’ve learnt from the internet.”	Stroke survivor	Appalasamy 2019
	Perceived benefit	“You have, every day, ten tablets ... (but) ... you don’t feel any better ... your arm is still without movement ... and [you think] ‘I’m taking tablets every day ... there is no point in taking it’”	HCP	Gibson 2021
		“‘I think aspirins are good for you. That’s the one I fancy. Well it thins the blood and the blood it flows and that stops any clots so I do like to take it. I just don’t see why I’m taking the other medication. I’m not fat or anything like that. I don’t get very high blood pressure and well cholesterol, what is cholesterol?”	Stroke survivor	Jamison 2018
**Practical barriers**	Cost	“if you’re, you’re not on a medical card ... you’d rack up a hefty bill quite quickly”.	HCP	Bell 2023
		“My gym program was set up by a physio from [community-based rehabilitation service]. He came with me the rest time, he set up a program, but if you're on $AU250 a week, you can't afford $AU14 a week to go to a gym, and $AU4 a day to go to a pool.”	Stroke survivor	Firth 2023
		“I bought some cheaper drugs instead of the expensive ones, but I never forgot to take medicine”	Stroke survivor	Lin 2022

		"And then there are people without health insurance, and they have even worse compliance…[However,] aspirin is affordable, it's cheap, and so they will use it. But metformin, statins, medication that costs more, they won't use it"	HCP	Xu 2021
	Physical health	". . . the (medication) packets are a pain in the neck. . . I'm one-handed initially, so I didn't use my left hand"	Stroke survivor	Gibson 2021
		"you've got to make sure they can read (the labels) ... before they get home ... you could have a hemianopia"	HCP	Gibson 2021
		"The things like dexterity—they might have been able to open blister packs before, they can't afterwards"	HCP	Bell 2023
		"if they have swallow issues, that's going to make things difficult ... sometimes you have to work around it"	HCP	Bell 2023
	Forgetfulness/Cognitive impairment	"I forget to go to the doctor to have the prescription renewed. I should have gone over a week ago."	Stroke survivor	Viprey 2020
		"So that has, that was also a shortcoming. That I noticed […] that I […] that I, that I forget a lot"	Stroke survivor	Kindermann 2023
		"I often forgot to take medicine. He [the patient's husband] usually put all the medicines on my table and reminded me to take them...If he also forgot, we would forget about it totally."	Stroke survivor	Gong 2019
		"I would like to take the medicines, but I inevitably forget to do it sometimes... It would be, of course, good if someone can remind me every day. "	Stroke survivor	Gong 2019
**Social support**	Role of family and friends	"My family helped me a lot. Especially [my family] in Turkey. […] I could, I say, really switch off, really. Not worry about anything."	Stroke survivor	Kindermann 2023

		"My wife sorts it out and that's why I don't know so much about it you see she [taps].She puts them there, I take them and that's it."	Stroke survivor	Jamison 2018
		"She has to take care of me. If she asks me to do exercise, I will do it. I have to do so. If not, she will not take care of me,"	Stroke survivor	Lin 2022
	Trust in HCP	"I think there's a bit of blind trust, with, with doctor…Doctor says, ©Oh, this does X Y Z and I will prescribe for you this (sic)"	Stroke Survivor	Firth 2023
		"So if the doctor says take ten pills a day, I'll, I'll do it…. he makes the decision and erm he, he's the boss man as you might say, who knows what he's up to"	Stroke survivor	Jamison 2018
		"He, my doctor, I trust him… blindly. So if he tells me to take something, I take it"	Stroke survivor	Viprey 2020
**Healthcare system factors**	Communication	"No. I didn't ask him, but he didn't tell me. Oh doctors… they're not like those we had in the past who explained everything to us, what we had, explained the medicine to us, what it would do. […] they don't have the time any more"	Stroke survivor	Viprey 2020
		"Sometimes, I don't understand what the doctor or pharmacist told me."	Stroke survivor	Appalasamy 2019
		"I know they (the doctors) are very busy. So they don't have time to explain."	Stroke survivor	Appalasamy 2019
	System-level influences	"I would probably feel additional resources, support or information would definitely help maximize my input into the patients holistically"	HCP	Bell 2023
		". . . we go through it [discharge summary] with the main carer. . . but we don't always have time to go through everything properly"	HCP	Gibson 2021

		“[Follow-up] is the difficult part of the process. If it were me, I wouldn’t go to the main hospital for a re-examination. It’s really very taxing”	HCP	Xu 2021
		“There isn’t any electronic network for the hospital system. The patient, if, for example, I give a prescription, and the other doctor wants to see it, the patient has to hand the paper prescription to the doctor.”	HCP	Xu 2021
**Psychological factors**	Mental health	“After suffering a stroke, besides the impact of the disease on their physical bodies, they may have psychological symptoms of depression or anxiety. They may also develop a sense of resignation or self-loathing towards their lives. They may not want to be treated.”	Stroke survivor	Xu 2021
		“So […] there was […] a medium depression […] on top of it, on top of the whole thing. […] and then mood swings. From […] just totally down in the dumps to[…] what one would call a normal mood.”	Stroke survivor	Kindermann 2023
		“And that has me of course also depressed, logically, because usually I am a smart woman, and that was then already depressing, uh, […]”	Stroke survivor	Kindermann 2023
	Motivation	“Their whole life’s changed and they’re really down in the dumps and they don’t want to live at all ... (so they think) ‘‘What’s the point in taking tablets?’’ ...”	HCP	Gibson 2021
		“What drives me is I still need a goal.”	Stroke survivor	Firth 2023
	Coping mechanisms	“I applied for this rehabilitation sport. I am trying to do rehabilitation sports and with this maybe […] I’ll get a kick, to say, with that, I can get rid of my fear.”	Stroke survivor	Kindermann 2023
		“It is hard to stop drinking, but I was sure I could do it. Several days later, how- ever, I found my blood sugar level increased, which made me upset. So, I just let it go, I began to drink again”	Stroke survivor	Lin 2022

	Emotional responses	"Obviously, there's different stages, there's different disabilities, post stroke ... But generally speaking, they're so overwhelmed by the change in their life that their input is minimal"	HCP	Bell 2023
		"Those who didn't experience stroke, don't understand how I feel"	Stroke survivor	Appalasamy 2019
**Medication characteristics**	Side effects	"I hadn't noticed being drowsy [while on a prescribed medication] but I was just worried about getting drowsy you know, because I wanted my license back and driving again…"	Stroke survivor	Firth 2023
		"Dizziness, I think, at least, that it [comes] from the medication- because before I never had something like that with other medications."	Stroke survivor	Kindermann 2023
		"Because of the medication [I] also [have] the tiredness, that's clear, but they also told me, that's because of the beta blocker. And, so [I suffer] the fatigue above all. Until the body gets used to it, I would say."	Stroke survivor	Kindermann 2023
	Medication-specific concerns	"they won't take a certain drug 1 day because they're like, I don't need that one, or I don't like the side effects that one gives me. And I feel like I get I feel more nauseous when I get this one or I feel more down, or I feel more out of sorts, more tired when I take that one".	HCP	Bell 2023
		"There's one of the two diuretics that I take practically never take because… it so…disrupts my life, having the urge to urinate every… every half-hour and that's … you really don't live well. And so, you have to stay at home."	Stroke survivor	Viprey 2020

Supplementary S4: Risk of Bias

	Gibson 2021	Hewitt 2024	Jamison 2018	DaCosta 2019	Bell 2023	White 2019	Viprey 2020	Kindermann 2023	Lin 2022	Xu 2020	Gong 2019	Appalasamy 2019	Firth 2023
**Categories of JBI Checklist**													
Congruity between stated philosophical perspective and research methodology?	☒	☒	☒	☒	☒	☒	☒	☒	☒	☒	☒	☒	☒
Congruity between research methodology and research question or objectives?	☒	☒	☒	☒	☒	☒	☒	☒	☒	☒	☒	☒	☒
Congruity between research methodology and methods used to collect data?	☒	☒	☒	☒	☒	☒	☒	☒	☒	☒	☒	☒	☒
Congruity between research methodology and representation and analysis of data?	☒	☒	☒	☒	☒	☒	☒	☒	☒	☒	☒	☒	☒
Congruity between research methodology and interpretation of results?	☒	☒	☒	☒	☒	☒	☒	☒	☒	☒	☒	☒	☒
Is there a statement locating the researcher culturally or theoretically?	☒	☐	☐	☐	☐	☐	☐	☐	☐	☐	☐	☐	☐
Is the influence of the researcher on the research, and vice-versa, addressed?	☐	☐	☒	☐	☐	☐	☐	☒	☐	☒	☐	☐	☒
Are participants, and their voices, adequately represented?	☒	☐	☒	☒	☒	☒	☒	☒	☒	☒	☒	☒	☒
Is the research ethical according to current criteria or, for recent studies is there evidence of ethical approval by an appropriate body?	☒	☒	☒	☒	☒	☒	☐	☐	☒	☒	☒	☒	☒
Do the conclusions drawn in the research report flow from the analysis, or interpretation, of the data?	☒	☒	☒	☒	☒	☒	☒	☒	☒	☒	☒	☒	☒

Supplementary S5: PRISMA Checklist

Section and Topic	Item #	Checklist item	Location where item is reported
**TITLE**			
Title	1	Identify the report as a systematic review.	Page 1
**ABSTRACT**			
Abstract	2	See the PRISMA 2020 for Abstracts checklist.	Page 1
**INTRODUCTION**			
Rationale	3	Describe the rationale for the review in the context of existing knowledge.	Page 2
Objectives	4	Provide an explicit statement of the objective(s) or question(s) the review addresses.	Page 2
**METHODS**			
Eligibility criteria	5	Specify the inclusion and exclusion criteria for the review and how studies were grouped for the syntheses.	Page 3
Information sources	6	Specify all databases, registers, websites, organisations, reference lists and other sources searched or consulted to identify studies. Specify the date when each source was last searched or consulted.	Page 3
Search strategy	7	Present the full search strategies for all databases, registers and websites, including any filters and limits used.	Appendix 1
Selection process	8	Specify the methods used to decide whether a study met the inclusion criteria of the review, including how many reviewers screened each record and each report retrieved, whether they worked independently, and if applicable, details of automation tools used in the process.	Page 3
Data collection process	9	Specify the methods used to collect data from reports, including how many reviewers collected data from each report, whether they worked independently, any processes for obtaining or confirming data from study investigators, and if applicable, details of automation tools used in the process.	Page 3
Data items	10a	List and define all outcomes for which data were sought. Specify whether all results that were compatible with each outcome domain in each study were sought (e.g. for all measures, time points, analyses), and if not, the methods used to decide which results to collect.	Page 3

Section and Topic	Item #	Checklist item	Location where item is reported
	10b	List and define all other variables for which data were sought (e.g. participant and intervention characteristics, funding sources). Describe any assumptions made about any missing or unclear information.	Page 3
Study risk of bias assessment	11	Specify the methods used to assess risk of bias in the included studies, including details of the tool(s) used, how many reviewers assessed each study and whether they worked independently, and if applicable, details of automation tools used in the process.	Page 4
Effect measures	12	Specify for each outcome the effect measure(s) (e.g. risk ratio, mean difference) used in the synthesis or presentation of results.	NA
Synthesis methods	13a	Describe the processes used to decide which studies were eligible for each synthesis (e.g. tabulating the study intervention characteristics and comparing against the planned groups for each synthesis (item #5)).	Pages 3 - 4
	13b	Describe any methods required to prepare the data for presentation or synthesis, such as handling of missing summary statistics, or data conversions.	NA
	13c	Describe any methods used to tabulate or visually display results of individual studies and syntheses.	Page 4
	13d	Describe any methods used to synthesize results and provide a rationale for the choice(s). If meta-analysis was performed, describe the model(s), method(s) to identify the presence and extent of statistical heterogeneity, and software package(s) used.	NA
	13e	Describe any methods used to explore possible causes of heterogeneity among study results (e.g. subgroup analysis, meta-regression).	NA
	13f	Describe any sensitivity analyses conducted to assess robustness of the synthesized results.	NA
Reporting bias assessment	14	Describe any methods used to assess risk of bias due to missing results in a synthesis (arising from reporting biases).	NA
Certainty assessment	15	Describe any methods used to assess certainty (or confidence) in the body of evidence for an outcome.	NA
**RESULTS**			
Study selection	16a	Describe the results of the search and selection process, from the number of records identified in the search to the number of studies included in the review, ideally using a flow diagram.	Page 4 and Fig. 1
	16b	Cite studies that might appear to meet the inclusion criteria, but which were excluded, and explain why they were excluded.	Fig. 1

Section and Topic	Item #	Checklist item	Location where item is reported
Study characteristics	17	Cite each included study and present its characteristics.	Table 1
Risk of bias in studies	18	Present assessments of risk of bias for each included study.	Appendix 4
Results of individual studies	19	For all outcomes, present, for each study: (a) summary statistics for each group (where appropriate) and (b) an effect estimate and its precision (e.g. confidence/credible interval), ideally using structured tables or plots.	NA
Results of syntheses	20a	For each synthesis, briefly summarise the characteristics and risk of bias among contributing studies.	Appendix 4 and Page 18
	20b	Present results of all statistical syntheses conducted. If meta-analysis was done, present for each the summary estimate and its precision (e.g. confidence/credible interval) and measures of statistical heterogeneity. If comparing groups, describe the direction of the effect.	Page 16 – 18, Fig 2 and Appendix 3
	20c	Present results of all investigations of possible causes of heterogeneity among study results.	NA
	20d	Present results of all sensitivity analyses conducted to assess the robustness of the synthesized results.	NA
Reporting biases	21	Present assessments of risk of bias due to missing results (arising from reporting biases) for each synthesis assessed.	NA
Certainty of evidence	22	Present assessments of certainty (or confidence) in the body of evidence for each outcome assessed.	NA
**DISCUSSION**			
Discussion	23a	Provide a general interpretation of the results in the context of other evidence.	18 – 20
	23b	Discuss any limitations of the evidence included in the review.	20 – 21

Section and Topic	Item #	Checklist item	Location where item is reported
	23c	Discuss any limitations of the review processes used.	20 – 21
	23d	Discuss implications of the results for practice, policy, and future research.	20
**OTHER INFORMATION**			
Registration and protocol	24a	Provide registration information for the review, including register name and registration number, or state that the review was not registered.	3
	24b	Indicate where the review protocol can be accessed, or state that a protocol was not prepared.	3
	24c	Describe and explain any amendments to information provided at registration or in the protocol.	3
Support	25	Describe sources of financial or non-financial support for the review, and the role of the funders or sponsors in the review.	20
Competing interests	26	Declare any competing interests of review authors.	21
Availability of data, code and other materials	27	Report which of the following are publicly available and where they can be found: template data collection forms; data extracted from included studies; data used for all analyses; analytic code; any other materials used in the review.	21
